# Alkylating agents activate an SLFN11-dependent vulnerability that confers PARP-1 inhibitor sensitivity in kidney cancer

**DOI:** 10.1186/s13046-026-03743-1

**Published:** 2026-06-09

**Authors:** Chenliang Wang, Ming Zhu, Lei Bao, Vanina Toffessi Tcheuyap, James Brugarolas, Weibo Luo, Yingfei Wang

**Affiliations:** 1 Department of Pathology, UT Southwestern Medical Center, Dallas, TX 75390 USA; 2 Kidney Cancer Program, Harold C. Simmons Comprehensive Cancer Center, UT Southwestern Medical Center, Dallas, TX 75390 USA; 3 Department of Internal Medicine, UT Southwestern Medical Center, Dallas, TX 75390 USA; 4 Department of Pharmacology, UT Southwestern Medical Center, Dallas, TX 75390 USA; 5 Department of Neurology, UT Southwestern Medical Center, Dallas, TX 75390 USA; 6 Peter O’Donnell Jr. Brain Institute, UT Southwestern Medical Center, Dallas, TX 75390 USA

**Keywords:** PARP-1, Alkylating agent, PARP inhibitor, ccRCC, SLFN11, BRCA1

## Abstract

**Background:**

PARP inhibitors have demonstrated notable clinical benefit, particularly in tumors with BRCA1/2 mutations. Clear cell renal cell carcinoma (ccRCC) carrying wild type (WT) BRCA1/2 is resistant to PARP inhibitor monotherapy. Strategies to enhance the sensitivity of BRCA1/2-WT ccRCC to PARP inhibitors remain challenging.

**Methods:**

An in vitro chemical screen was performed to identify alkylating agents that synergize with PARP inhibition using cell viability assays. ccRCC sensitivity to individual alkylating agents was further assessed by measuring PARP-1 activity. The therapeutic effects of monotherapy and combination treatment with alkylating agents and PARP inhibitors were evaluated through DNA damage repair assays, cell viability assays, colony formation assays, ccRCC cell xenograft assays, and ccRCC patient-derived xenograft assays. Mechanistic insights and DNA repair pathway involvement were assessed using comparative expression profiling and gain- and loss-of-function approaches.

**Results:**

BRCA1/2-WT ccRCC exhibits markedly higher PARP-1 activity compared to breast cancers. A subset of alkylating agents, including Temozolomide (TMZ) and Streptozocin, is identified to induce hyperactivation of PARP-1, creating a PARP-1 dependency in ccRCC cells that renders these cells sensitive to PARP inhibitors in vitro and in mice. Mechanistically, SLFN11 is selectively upregulated and boosts proteasomal degradation of BRCA1 protein in ccRCC cells. Loss of SLFN11 increases BRCA1 levels and enhances homologous recombination repair, mitigating prolonged excessive DNA damage in ccRCC cells following combination treatment with TMZ and Olaparib. SLFN11 is inversely correlated with BRCA1 expression and potentiates the therapeutic efficacy of TMZ and Olaparib combination treatment in ccRCC patient-derived xenograft models.

**Conclusion:**

Our findings reveal an intrinsic SLFN11-dependent vulnerability in ccRCC that synergizes with alkylating agents to induce an acquired PARP-1 dependency, thereby sensitizing BRCA1/2-WT tumors to PARP inhibition. Therefore, this work uncovers a potential therapeutic strategy for targeting SLFN11-high kidney cancers.

**Supplementary Information:**

The online version contains supplementary material available at 10.1186/s13046-026-03743-1.

## Background

Synthetic lethality was first described in *Drosophila melanogaster* genetic studies in 1922 [1], and later enabled the identification of mutant breast cancer gene 1 and 2 (BRCA1/2) as synthetic lethal partners with poly(ADP-ribose) polymerase (PARP) inhibition in breast cancer [2, 3]. This synthetic lethality strategy has been developed to treat germline BRCA-mutant ovarian cancer, HER2-negative breast cancer, as well as metastatic pancreatic and prostate cancers [4–6]. To date, four PARP inhibitors, including Olaparib, Rucaparib, Niraparib and Talazoparib, have been approved by the FDA for the treatment of BRCA-mutant cancers, demonstrating notable clinical benefit [7]. Accumulating studies have shown that mutations in homologous recombination (HR) repair proteins beyond BRCA1/2, a phenomenon called BRCAness, can also confer the sensitivity of cancer cells to PARP inhibitors [8, 9], which broadens the potential application of PARP inhibitors across a wide range of cancers. Moreover, another recent bioinformatics study implicated a wide presence of BRCAness across human BRCA-proficient cancers, including clear cell renal cell carcinoma (ccRCC) [10], which is the most common subtype (~ 80%) of kidney cancer featured with loss of the tumor suppressor gene *VHL* (von Hippel-Lindau) [11]. However, ccRCC cells respond poorly to single-agent PARP inhibitor treatment in preclinical models [12], suggesting that the previously characterized BRCAness feature is not sufficient to render ccRCC cells vulnerable to PARP inhibitors. Although targeted inhibitors of angiogenesis, epigenetic reprogramming, and oncogenic kinase pathways have been proposed to induce HR deficiency in tumors [13], sensitizing BRCA1/2-wild type (WT) tumors and rendering these previously deemed ineligible tumors susceptible to PARP inhibitors remain a major challenge.

Alkylating agents represent the earliest and currently most prevalent chemotherapy drugs for cancer treatment in the clinic [14–16]. Five traditional categories of alkylating agents, including nitrogen mustard, nitrosoureas, alkyl sulfonates, triazines and ethylenimines, and other non-classical alkylating-like agents [14], add an alkyl group on DNA and cause DNA cross-linking, abnormal base pairing, or DNA strand breaks [17]. These DNA lesions are primarily repaired by direct base repair, base excision repair (BER), or nucleotide excision repair (NER) [17]. Failure to repair DNA damage induced by alkylating agents results in cell death. We and others have shown that treatment with alkylating agents *N*-Methyl-*N*′-nitro-*N*-nitrosoguanidine (MNNG) and methyl methanesulfonate (MMS) can specifically activate PARP-1 in cancer cells [18, 19]. PARP-1, as a DNA damage sensor [20], senses DNA damage and utilizes NAD^+^ as the substrate to catalyze the addition of poly-ADP-ribose (PAR) toward different acceptor proteins, including PARP-1 itself [21–23]. As a result, repair proteins and nucleases are recruited to sites of DNA damage, thereby facilitating DNA repair [24]. Inhibition of PARP-1 impairs DNA single-strand break repair and eventually causes DNA double-strand breaks, which are primarily repaired through either HR or non-homologous end joining (NHEJ) mechanism [20]. It remains largely unknown whether alkylating agents trigger PARP dependence to render BRCA1/2-WT cancer cells vulnerable to PARP inhibitors.

Schlafen family member 11 (SLFN11), a DNA/RNA helicase-like protein inactivated in about 45% cancer cells [25], has emerged as a key determinant of cellular responses to DNA-damaging agents. High expression of SLFN11 has been suggested to correlate with the sensitivity of human small-cell lung cancer to DNA-damaging therapies [26, 27]. Multiple mechanisms have been proposed for the function of SLFN11 in cancer cells, including promoting stalled replication fork degradation [28], cleavage of type II tRNAs [29], and destabilizing the RPA-ssDNA complex [30], thereby modulating the cellular response to DNA damage and replication stress. Together, these accumulating lines of evidence underscore a critical role of SLFN11 in the DNA damage response. Interestingly, although exploratory clinical analyses in small-cell lung cancer have implicated SLFN11 as a predictor of response to Temozolomide (TMZ) plus PARP inhibitor [26, 27], analysis of the CellMinerCDB-GDSC dataset shows no measurable correlation between SLFN11 and TMZ monotherapy sensitivity in lung cancer cell lines and only a weak correlation across human cancer lines (https://discover.nci.nih.gov/cellminercdb/). This discrepancy highlights a key conceptual gap: how SLFN11 confers actionable therapeutic vulnerability to PARP inhibitors in BRCA1/2-WT cancers and the specific treatment contexts in which this SLFN11-dependent state develops.

In this study, we reveal elevated PARP-1 activity and high SLFN11 expression as two key features of ccRCC. SLFN11 functions as an intrinsic negative regulator of BRCA1 protein expression in ccRCC cells. We also identify a subgroup of alkylating agents including TMZ and Streptozocin (STZ) that trigger PARP-1 dependency in SLFN11-high ccRCC and sensitize these cells to PARP inhibitors in vitro and in mice. Thus, the combination treatment with TMZ and PARP inhibitor synergistically induces sustained DNA damage and cell death in ccRCC, and profoundly suppresses the growth of patient-derived xenografts (PDXs). Collectively, these findings reveal an effective therapeutic strategy and a biomarker for the combination treatment in BRCA1/2-WT ccRCC.

## Methods

### Plasmid constructs

Full-length human SLFN11 cDNA was amplified by PCR with specific primers (Supplementary Table 2) and subcloned into pLVX-Ubc-FLAG-N or pcDNA3.1-V5 vector. Full-length human BRCA1 cDNA was subcloned into p3xFLAG-CMV-7 vector from pMH-SFB-BRCA1 (Addgene, #99394). DNA oligonucleotides of the single-guide RNA targeting human PARP-1 or SLFN11 (Supplementary Table 2) were annealed and ligated into BsmBI-linearized lentiCRISPRv2 vector (Addgene, #52961). All recombinant plasmids were verified by Sanger sequencing.

### Cell culture and knockout (KO) cell line generation

RCC10, 786-O, RCC4, SUM159, and MDA-MB-231 cells were cultured in DMEM with 10% heat-inactivated fetal bovine serum (FBS) at 37 °C in a 5% CO2/95% air incubator. UMRC2 cells were cultured in MEM with 1x GlutaMAX, 1% non-essential amino acids, and 10% heat-inactivated FBS. PARP1-KO and SLFN11-KO cells were generated with the CRISPR/Cas9 technique as described previously [31] and selected with puromycin (1 µg/mL). The resulting polyclonal cell populations were used without single-cell selection, and gene KO was verified by immunoblotting. All cell lines were tested by short tandem repeat DNA profiling analysis and mycoplasma-free.

### In vitro chemical screens

RCC10 and RCC10-PARP-1-KO cells (1 × 10^5^/well) were seeded onto 12-well plates for 16 h. Cells were treated for 24–96 h with vehicle or drugs/chemicals at the dose shown in Supplementary Table 1. Cells were stained with propidium iodide (PI) (2 µM) and Hoechst 33342 (7 µM, ThermoFisher) for 10 min and imaged under a Zeiss Observer Z1 microscope. The numbers of total and PI-positive cells were quantified as described previously [18].

### Colony formation assay

Cells (200 cells/well) were seeded on a 12-well plate and treated with indicated chemicals for 10 days. Colonies were washed with phosphate-buffered saline (PBS), fixed with 100% methanol, and stained with 0.01% crystal violet. The crystal violet dye was dissolved in 10% acetic acid and measured at optical density 570 nm in a Tecan Spark 10M plate reader.

### Cell death assay

1 × 10^5^ cells/well were seeded on a 12-well plate for 16 h, followed by the treatment with MNNG (30 µM, 15 min) or TMZ (500 µM, 48 h) in the presence or absence of PARP inhibitors. After 15 min of treatment with MNNG, cells were changed to fresh medium with or without indicated PARP inhibitors for continuous culture for 24 h. Cells were stained with PI (2 µM) and Hoechst 33342 (7 µM, ThermoFisher) for 10 min and imaged under a Zeiss Observer Z1 microscope. The numbers of total and PI-positive cells were quantified as described previously [18].

### Immunoblot assay

Cells were lysed with NETN lysis buffer (150 mM NaCl, 1 mM EDTA, 10 mM Tris-HCl, pH 8.0, 0.5% NP-40 and protease inhibitor cocktail), followed by sonication for 15 s. The equal amount of proteins were separated on SDS-PAGE gel and transferred to nitrocellulose membrane. The membrane was blocked with 5% milk and incubated overnight with primary antibodies (Supplementary Table 3), followed by HRP-conjugated secondary antibodies. Proteins were visualized by chemiluminescence with ECL Prime (GE Healthcare).

### Immunostaining assay

Cells (1 × 10^5^ cells/well) were seeded onto glass coverslips placed in a 12-well plate. Next day, cells were treated with TMZ (500 µM) with or without Olaparib (10 µM) for indicated time, fixed with 4% paraformaldehyde, permeabilized with 0.05% Triton X-100, and blocked with 5% BSA in PBS. For RPA2 and RAD51 staining, cells were first treated with the buffer (20 mM HEPES, 50 mM NaCl, 3 mM MgCl_2_, 0.3 M sucrose, 0.2% Triton X-100) on ice for 5 min to remove soluble nuclear proteins. Then cells were fixed and permeabilized as described above. Next, cells were incubated overnight with anti-γH2AX antibody (1:300), anti-RPA2 antibody (1:300), anti-RAD51 antibody (1:300) at 4^ο^C, washed with PBS containing 0.1% Tween-20 for 3 times, and incubated with Alexa488 donkey anti-mouse IgG (1:1,000) or Cy3 donkey anti-rabbit IgG (1:1,000) for 90–120 min in dark. After washing three times, cells were incubated with DAPI (1:1,000) for 10 min. Cells were washed again and mounted with anti-fade mounting medium. Immunofluorescent analysis was carried out with a Zeiss Observer Z1 fluorescence microscope.

### Quantitative Reverse Transcription-Polymerase Chain Reaction (qRT-PCR) assay

Total RNA was isolated using TRIzol reagent (ThermoFisher) and cleaned with RNase-free DNase I (ThermoFisher). cDNA was synthesized using the iScript cDNA synthesis kit (Bio-Rad). Real-time PCR was performed by a CFX-96 real-time system (Bio-Rad) using iTaq universal SYBR green supermix (Bio-Rad) with primers listed in Supplementary Table 4. mRNA expression was quantified as described previously [18].

### Pulsed-field gel electrophoresis

Pulsed-field gel electrophoresis was performed as described previously [32]. Briefly, scrambled control (SC) or SLFN11-KO RCC10 cells were treated with TMZ (500 µM) in the presence or absence of Olaparib for 6 h. Cells were washed with cold PBS twice, and then lysed with proteinase K (100 µg/mL) in lysis buffer (50 mM Tris/HCl, pH 8.0, 100 mM NaCl, 5 mM EDTA, 2 mM CaCl_2_, 1% SDS) at 55 °C for 12 h. Genomic DNA was extracted and immediately separated on a 1.2% pulsed-field agarose in 0.5 × TBE buffer with initial switch time of 1.5 s and a final switch time of 3.5 s for 12 h at 6 V/cm. The gel was then stained with 0.5 µg/mL ethidium bromide and imaged using Bio-Rad ChemiDoc touch imaging system. Uncleaved genomic DNA was quantified with Image J software.

### Animal studies

Animal studies were approved and conducted under the oversight of the UT Southwestern Institutional Animal Care and Use Committee. Human ccRCC PDX tumors were approved by the Institutional Review Board at UT Southwestern Medical Center with written informed consent and generated as described previously [33]. 1 × 10^6^ UMRC2 cells suspended in PBS/Matrigel (1:1, Corning) or PDX tumors (2 × 2 × 2 mm^3^) were implanted subcutaneously into the right flank of male NOD-SCID-IL2RGKO (NSG) mice. Mice were intraperitoneally administered with vehicle, TMZ or STZ (5 mg/kg, daily for 15 days, with 2 days of rest for every 5 days-treatment), and/or Olaparib (50 mg/kg, daily for 15 days, with 2 days of rest in-between every 5 days-treatment), once the tumor volume reached 100 mm^3^. Tumor volume was measured as described previously [31]. 21 days after drug treatment, tumors were harvested, imaged, and weighed.

### Statistical analysis

Statistical analysis was performed by two-tailed Student’s *t* test between two groups, and one-way or two-way ANOVA with multiple testing correction within multiple groups. Data were expressed as mean ± SEM. A *p* < 0.05 is considered significant.

## Results

### A targeted drug screen identifies a group of alkylating agents that trigger a PARP-1 dependency in ccRCC cells

**Fig. 1:**
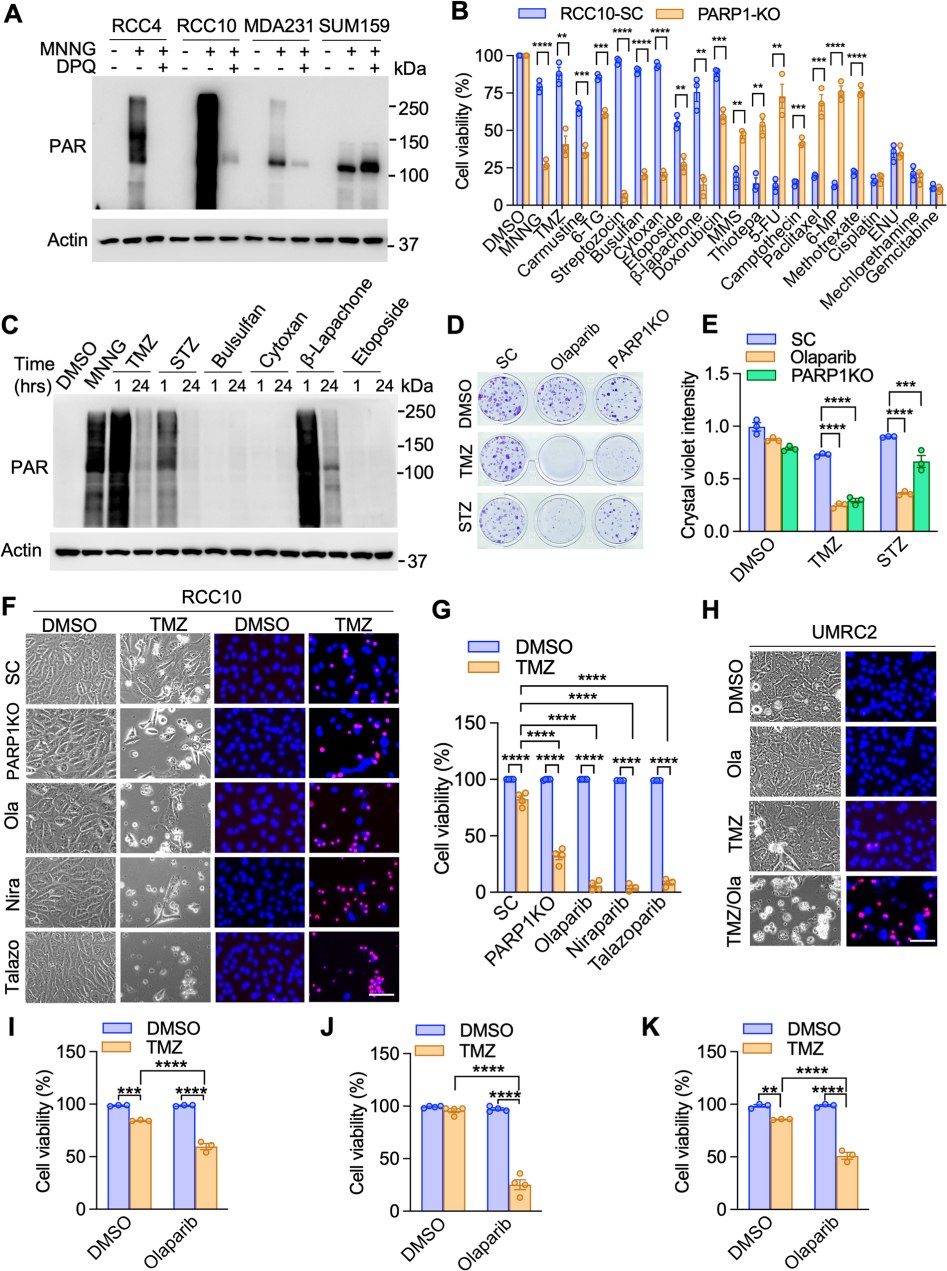
Identification of alkylating agents that elicit a PARP-1 dependency in ccRCC cells. **A** Immunoblot analysis of PARP-1 activation in various cancer cells 15 min after treatment with MNNG (50 µM) in the presence or absence of PARP-1 inhibitor DPQ (30 µM). **B** A cell viability screen in parental and PARP1-KO RCC10 cells treated with indicated compounds for 24–96 h (mean ± SEM, *n* = 3). Treatment durations were selected based on the known kinetics of each drug to capture peak cytotoxic effects. **P < 0.01; ***P < 0.001; ****P < 0.0001 by 2-tailed Student’s *t* test. **C** Immunoblot analysis of PARP-1 activation in RCC10 cells treated with indicated compounds for 0.25, 1, or 24 h. **D** and **E** Representative colony survival in Scrambled control (SC) and PARP1-KO RCC10 cells treated with vehicle, TMZ (500 µM), or STZ (500 µM) for 10 days and RCC10 cells treated with Olaparib (10 µM) and/or TMZ (500 µM) or STZ (500 µM) for 10 days (**D**). The intensity of crystal violet is quantified in **E** (mean ± SEM, *n* = 3). ***P < 0.001; ****P < 0.0001 by two-way ANOVA with Tukey’s multiple comparisons test. **F** and **G** Representative cell death images in SC and PARP1-KO RCC10 cells treated with vehicle or TMZ (500 µM) and RCC10 cells treated with TMZ (500 µM) and/or Olaparib (Ola, 10 µM), Niraparib (Nira, 1 µM), or Talazoparib (Talazo, 10 nM) (**F**). The percentage of PI-positive cells (red) is quantified in **G** (mean ± SEM, *n* = 3). ****P < 0.0001 by two-way ANOVA with Tukey’s multiple comparisons test. Scale bar, 100 μm. **H** and **I** Representative cell death images in UMRC2 cells treated with TMZ (500 µM) and/or Olaparib (10 µM) (**H**). The percentage of PI-positive cells (red) is quantified in **I** (mean ± SEM, *n* = 3). ***P < 0.001; ****P < 0.0001 by two-way ANOVA with Tukey’s multiple comparisons test. Scale bar, 100 μm. **J** and **K** Cell death assay in RCC4 (J, mean ± SEM, *n* = 4) and 786-O (K, mean ± SEM, *n* = 3) cells treated with TMZ (500 µM) and/or Olaparib (10 µM). **P < 0.01; ****P < 0.0001 by two-way ANOVA with Tukey’s multiple comparisons test

To determine PARP-1 activity in BRCA1/2-WT cancer cells that are resistant to single-agent PARP inhibitors, we treated two ccRCC cell lines (RCC4 and RCC10) and two triple-negative breast cancer cell lines (MDA-MB-231 and SUM159) with vehicle or a specific PARP-1 activation inducer MNNG (50 µM) for 15 min. Both RCC4 and RCC10 cells exhibited much stronger PARP-1 activity than MDA-MB-231 and SUM159 cells, as determined by PAR formation upon MNNG treatment under the same conditions (Fig. 1A). PARP-1 expression levels were comparable among all these cell lines (Fig. S1A). PARP-1 KO or pre-treatment with a PARP inhibitor DPQ (30 µM) fully blocked MNNG-induced PARP-1 activation (Fig. 1A and Fig. S1B and C). These results indicate that ccRCC cells display a hyper-activity of PARP-1 in response to alkylating agent compared with breast cancer cells.

We next conducted a targeted drug screen of 20 clinically approved or clinically tested compounds, including various alkylating agents and other common chemotherapy drugs in isogenic RCC10 cells and PARP-1-KO counterparts (Supplementary Table 1). The goal was to identify agents that induce a PARP-1 dependency and selectively kill PARP-1-KO cells, using PI staining as a cell death readout. MNNG (30 µM) served as a positive control, which showed a selective killing effect when combined with PARP-1 KO or Olaparib (10 µM) in RCC10 cells (Fig. S1D and E). As shown in Fig. 1B, PARP-1-KO RCC10 cells showed enhanced sensitivity to a subgroup of alkylating agents, including TMZ (1 mM), STZ (1 mM), Busulfan (200 µM), and Cyclophosphamide (also known as Cytoxan, 1 mM), and other compounds including topoisomerase inhibitor Etoposide (6 µM) and β-Lapachone (4 µM) as compared with parental RCC10 cells (cell viability ratio of RCC10/PARP-1-KO > 2). In contrast, MMS (4 mM), Thiotepa (50 µM), Fluorouracil (5-FU, 200 µM), Camptothecin (500 nM), Paclitaxel (100 nM), Mercaptopurine (6-MP, 200 µM), and Methotrexate (400 µM) were shown to preferably kill parental RCC10 cells (cell viability ratio of RCC10/PARP-1-KO < 0.5). Other chemicals/drugs we tested, including Carmustine (200 µM), 6-Thioguanine (6-TG, 300 µM), Doxorubicin (400 nM), Cisplatin (20 µM), N-ethyl-N-nitrosourea (ENU, 10 mM), Mechlorethamine (20 µM), and Gemcitabine (500 nM) did not show a selective killing effect between RCC10 cells and PARP-1-KO counterparts (cell viability ratio of RCC10/PARP-1-KO between 0.5 and 2). Next, the effects of TMZ, STZ, Busulfan, Cytoxan, Etoposide, and β-Lapachone on PARP-1 activation were evaluated in RCC10 cells at 1 h and 24 h via measuring PAR signal intensity and duration. Among these candidates, TMZ (1 mM) and β-Lapachone (200 µM) induced stronger and more prolonged PARP-1 activation than the other treatments (Fig. 1C). STZ (1.5 mM) also robustly induced PARP-1 activation in ccRCC cells but the response was relatively short-lived (Fig. 1C). Previous studies have shown that PARP-1 inhibition protects cancer cells against β-Lapachone [34, 35]. Thus, TMZ and STZ were chosen as representative drugs for further studies.

To validate our screen results, we performed clonogenic assay in SC and PARP-1-KO RCC10 cells after treatment with the different doses of TMZ. We found that TMZ at the doses of 0.25 and 0.5 mM significantly enhanced cell death in PARP-1-KO cells compared with SC cells (Fig. 1D and E, S1F and G). STZ (0.5 mM) also increased cell death in PARP-1-KO RCC10 cells to a lesser degree (Fig. 1D and E). Next, we studied whether TMZ increases sensitivity of ccRCC cells to PARP inhibitors. To this end, RCC10 cells were treated with DMSO or TMZ (0.5 mM) with or without Olaparib (10 µM), Niraparib (1 µM), or Talazoparib (10 nM). In line with PARP-1 KO, all three PARP inhibitors robustly killed RCC10 cells when combined with TMZ (Fig. 1F and G). Similar results were observed in other ccRCC cell lines, including UMRC2, RCC4, and 786-O, when they were treated with TMZ and Olaparib (Fig. 1H-K). These cancer cells did not respond to the single treatment with TMZ or PARP inhibitors (Fig. 1F and G). TMZ and Olaparib combination treatment killed RCC10 and RCC10-VHL cells to a similar extent (Fig. S1H and I), suggesting that VHL is dispensable for the efficacy of TMZ and Olaparib combination treatment. Collectively, these findings identify that TMZ and STZ trigger a PARP-1 dependency that renders ccRCC cells vulnerable to PARP inhibitors.

### Alkylating agents increase the sensitivity of ccRCC to PARP inhibitor in vivo

**Fig. 2:**
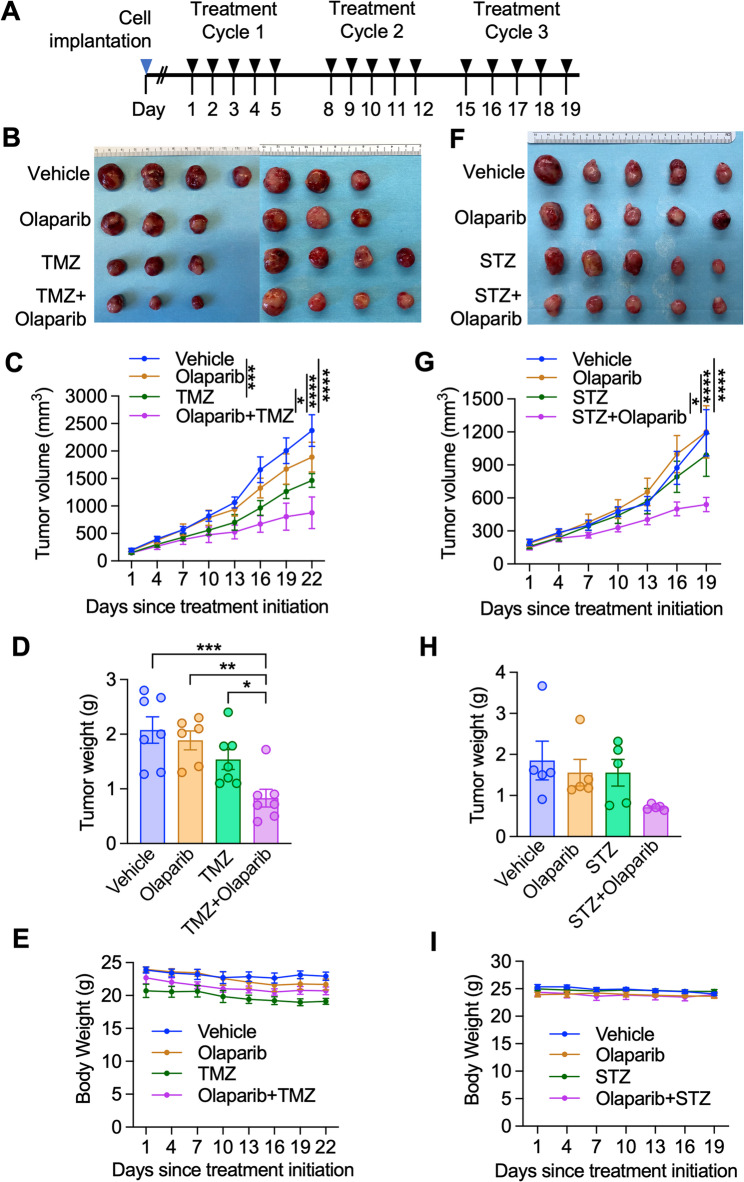
Alkylating agent and PARP inhibitor synergistically inhibit ccRCC growth in vivo. **A** Schematic of the treatment regimen in mice. **B**-**E** UMRC2 cells were subcutaneously implanted into male NSG mice and mice were administered vehicle, Olaparib (50 mg/kg), and/or TMZ (5 mg/kg). Tumor images from two independent studies (**B**), tumor volume (**C**), tumor weight (**D**) and mouse body weight after treatments (**E**) are shown (mean ± SEM, *n* = 6–7). *P < 0.05; **P < 0.01; ***P < 0.001; ****P < 0.0001 by two-way ANOVA with Tukey’s multiple comparisons test (**C**) or one-way ANOVA with Dunnett’s multiple comparisons test (**D**). **F**-**I** UMRC2 cells were subcutaneously implanted into male NSG mice and mice were administered vehicle, Olaparib (50 mg/kg), and/or STZ (5 mg/kg). Tumor images (**F**), tumor volume (**G**), tumor weight (**H**) and mouse body weight after treatments (**I**) are shown (mean ± SEM, *n* = 5). *P < 0.05; ****P < 0.0001 by two-way ANOVA with Tukey’s multiple comparisons test (**G**)

We next studied whether TMZ or STZ increases the sensitivity of ccRCC to Olaparib in vivo. To this end, UMRC2 cells were subcutaneously implanted into the flank of male NSG mice. Once the tumor volume reached about 100 mm^3^, mice were randomly stratified into four groups and treated with 3 cycles of vehicle, TMZ (5 mg/kg), Olaparib (50 mg/kg), or combination of TMZ and Olaparib (Fig. 2A). The single treatment with TMZ and Olaparib had a modest effect on tumor growth inhibition in mice, however, the combination of Olaparib and TMZ synergistically inhibited tumor growth in mice (Fig. 2B-D). All treatments had no effect on mouse body weight (Fig. 2E), suggesting little toxicity of these single or combined treatments at their doses we tested. Similarly, combination treatment with STZ (5 mg/kg) and Olaparib synergistically inhibited xenograft tumor growth, whereas single-agent treatment had no obvious effect (Fig. 2F-I). These findings indicate that TMZ and STZ sensitize ccRCC tumors to Olaparib in a mouse model.

### Genetic or pharmacological inhibition of PARP-1 in combination with TMZ promotes prolonged DNA damage in ccRCC cells

**Fig. 3:**
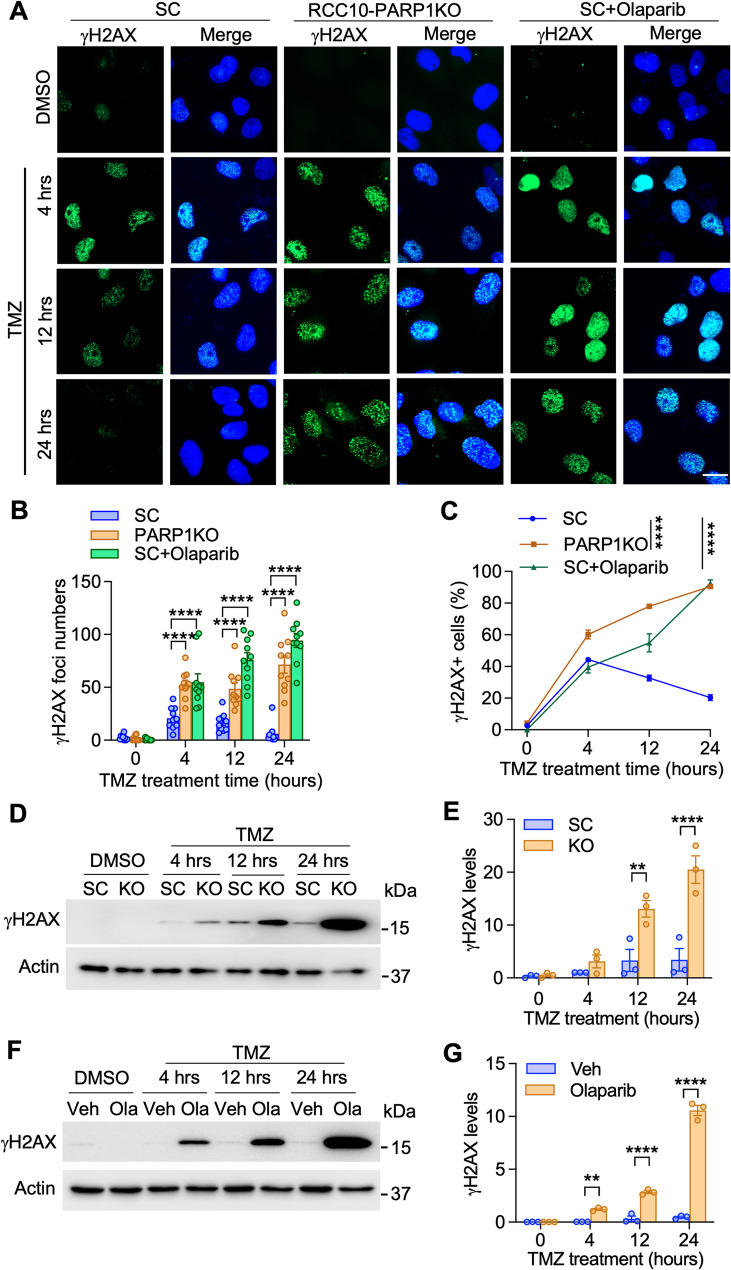
Alkylating agent and PARP inhibitor combination treatment induces prolonged DNA damage in ccRCC cells. **A**-**C** Representative γH2AX staining images in SC and PARP1-KO RCC10 cells treated with TMZ (500 µM) for indicated time and in RCC10-SC cells treated with TMZ (500 µM) and/or Olaparib (10 µM) for indicated time (**A**). The numbers of γH2AX foci per cell are quantified in B (mean ± SEM, *n* = 10). The percentage of γH2AX-positive cells is quantified in **C** (mean ± SEM, *n* = 6). Scale bar, 20 μm. ****P < 0.0001 by two-way ANOVA with Tukey’s multiple comparisons test. **D** and **E** Immunoblot analysis of γH2AX in SC and PARP1-KO RCC10 cells treated with vehicle or TMZ (500 µM) for indicated time. **F** and **G** Immunoblot analysis of γH2AX in RCC10 cells treated with TMZ (500 µM) and/or Olaparib (Ola, 10 µM) for indicated time. Veh, vehicle. **P < 0.01; ****P < 0.0001 by two-way ANOVA with Sidak’s multiple comparisons test

TMZ is known to induce DNA damage in the cell, and PARP-1 is a DNA damage sensor that plays a critical role in recruiting DNA repair machinery [20]. Thus, the effects of TMZ (0.5 mM) and Olaparib (10 µM) treatment on DNA damage repair were assessed in parental RCC10 cells and PARP-1 KO counterparts. We found that TMZ induced foci of γH2AX, a marker of DNA damage, in RCC10 cells 4 h after treatment, which then declined at 12 h and 24 h in a time-dependent manner (Fig. 3A-C). Genetic deletion and pharmacological inhibition of PARP-1 by Olaparib both inhibited DNA repair and enhanced accumulation of excessive γH2AX levels in RCC10 cells, even 24 h after TMZ treatment (Fig. 3A-C). Similarly, immunoblot analysis further supported that combination of TMZ and PARP inhibitor, as well as TMZ combined with PARP-1 deletion, led to increased accumulation of DNA damage in ccRCC cells in a time-dependent manner (4–24 h) compared with single-agent treatments (Fig. 3D-G). These findings indicate that genetic or pharmacological inhibition of PARP-1 synergizes with TMZ to induce prolonged and excessive DNA damage in ccRCC cells.

### Identification of SLFN11 as a regulator of alkylating agent-induced sensitivity to PARP inhibitor in ccRCC cells

**Fig. 4:**
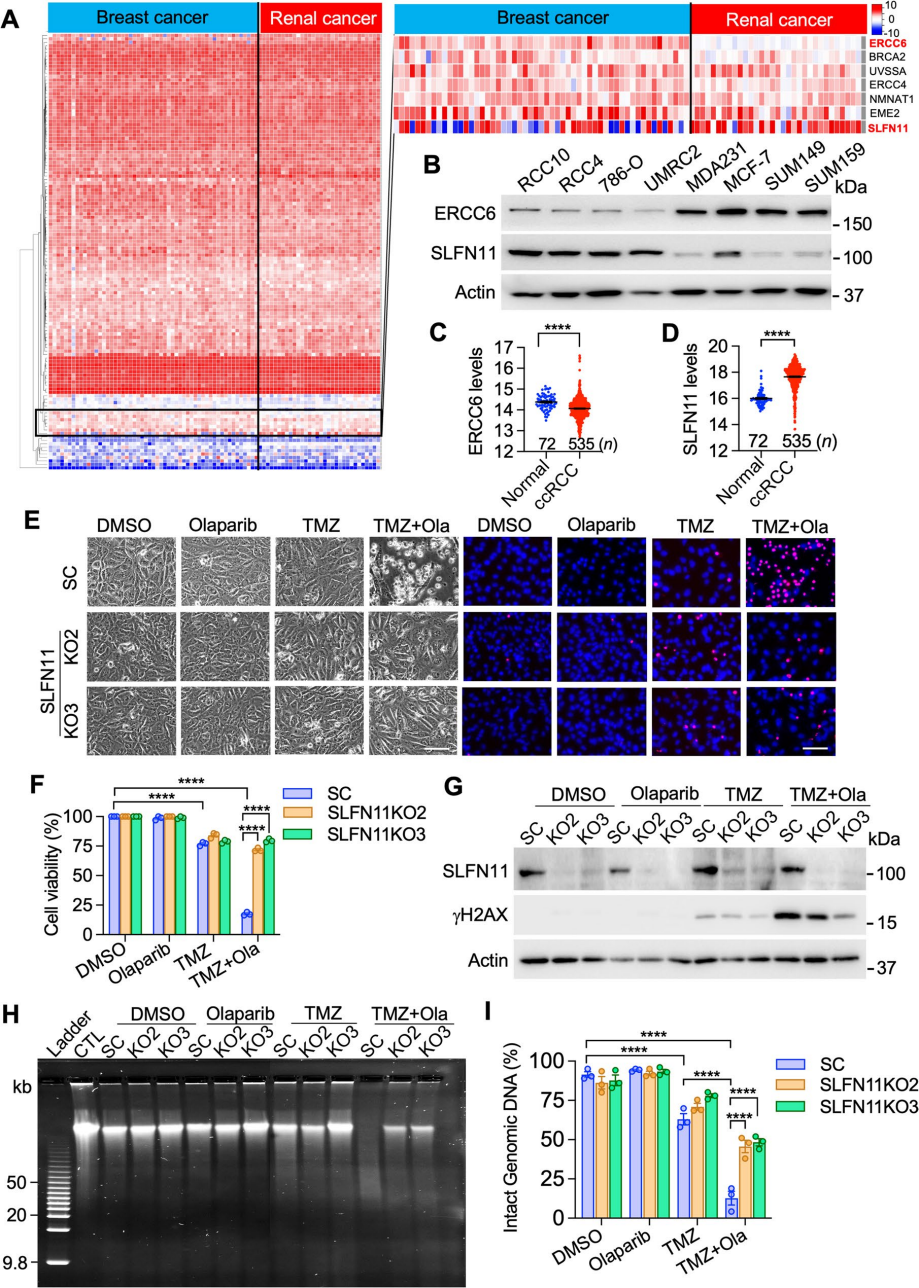
Identification of SLFN11 as a key regulator of alkylating agent-induced vulnerability to PARP inhibitor in ccRCC cells. **A** Heatmap of DNA damage repair protein expression in kidney and breast cancer cell lines. Data were retrieved from Cancer Cell Line Encyclopedia. **B** Immunoblot analysis of SLFN11 and ERCC6 expression in various kidney and breast cancer cell lines. **C** and **D** *ERCC6* (**C**) and *SLFN11* (**D**) mRNAs in normal kidney tissues and ccRCC tumors. Data were retrieved from TCGA. ****P < 0.0001 by 2-tailed Student’s *t* test. **E** and **F** Representative cell death images in SC and SLFN11-KO RCC10 cells 24 h after treatment with TMZ (500 µM) and/or Olaparib (10 µM) (**E**). The percentage of PI-positive cells (red) is quantified in F (mean ± SEM, *n* = 3). Scale bar, 100 μm. ****P < 0.0001 by two-way ANOVA with Tukey’s multiple comparisons test. **G** Immunoblot analysis of SLFN11 and γH2AX in SC and SLFN11-KO RCC10 cells treated with TMZ (500 µM) and/or Olaparib (10 µM) for 24 h. **H** and **I** Pulsed-field gel electrophoresis assay in SC and SLFN11-KO RCC10 cells 6 h after treatment with TMZ (500 µM) and/or Olaparib (10 µM) (H). The percentage of uncleaved genomic DNA is quantified in I (mean ± SEM, *n* = 3). ****P < 0.0001 by two-way ANOVA with Tukey’s multiple comparisons test. CTL, control genomic DNA from naïve cells

Next, we studied whether DNA repair pathways are functionally impaired in ccRCC cells and how excessive DNA damage occurs upon combined treatment with TMZ and Olaparib. To this end, we analyzed the mRNA expression of 127 DNA repair genes in 32 renal cancer cell lines from the Cancer Cell Line Encyclopedia with 55 breast cancer cell lines served as controls. We found that PARP-1 KO or Olaparib fully blocked MNNG-induced death of breast cancer MDA-MB-231 cells (Fig. S2A and B), which was opposite to ccRCC cells (Fig. 1F-K, Fig. S1D and E). Of these DNA repair genes, SLFN11 was highly expressed in renal cancer cell lines but showed relatively weak expression in breast cancer cells (Fig. 4A). In contrast, ERCC6 was expressed at low levels in renal cancer cell lines but was highly expressed in breast cancer cells (Fig. 4A). The selective expression of ERCC6 and SLFN11 proteins was confirmed in breast cancer cell lines and ccRCC cell lines, respectively (Fig. 4B, Fig. S3A and B). We further analyzed transcriptome in ccRCC from The Cancer Genome Atlas and found significant upregulation of *SLFN11* mRNA and downregulation of *ERCC6* mRNA in tumor cells as compared with adjacent normal kidney tissues (Fig. 4C and D).

To determine the role of SLFN11 in alkylating agent-induced sensitivity to PARP inhibitor in ccRCC, we generated two independent SLFN11-null RCC10 cell lines using the CRISPR/Cas9 technique (Fig. 4E). These KO cells and SC counterparts were treated with TMZ (0.5 mM) and/or Olaparib (10 µM) for 2 days. SLFN11 KO2 or KO3 robustly blocked TMZ + Olaparib combination-induced cell death (Fig. 4E and F). Similar results were found in RCC10 cells treated with MNNG and/or Olaparib (Fig. S4A and B) and UMRC2 cells treated with TMZ and/or Olaparib (Fig. S4C-D). Notably, SLFN11 KO2 or KO3 blocked γH2AX induction by TMZ or MNNG combined with Olaparib in RCC10 and UMRC2 cells (Fig. 4G, Fig. S5A-H). Pulse-field gel electrophoresis analysis further confirmed DNA damage by TMZ + Olaparib combination treatment but not their individual treatment in RCC10 cells, which was blocked by SLFN11 KO2 or KO3 (Fig. 4H and I). These findings indicate that SLFN11 is upregulated and required for TMZ + Olaparib combination-induced cell death in ccRCC.

### SLFN11 promotes ubiquitination and degradation of BRCA1 protein in ccRCC cells

**Fig. 5:**
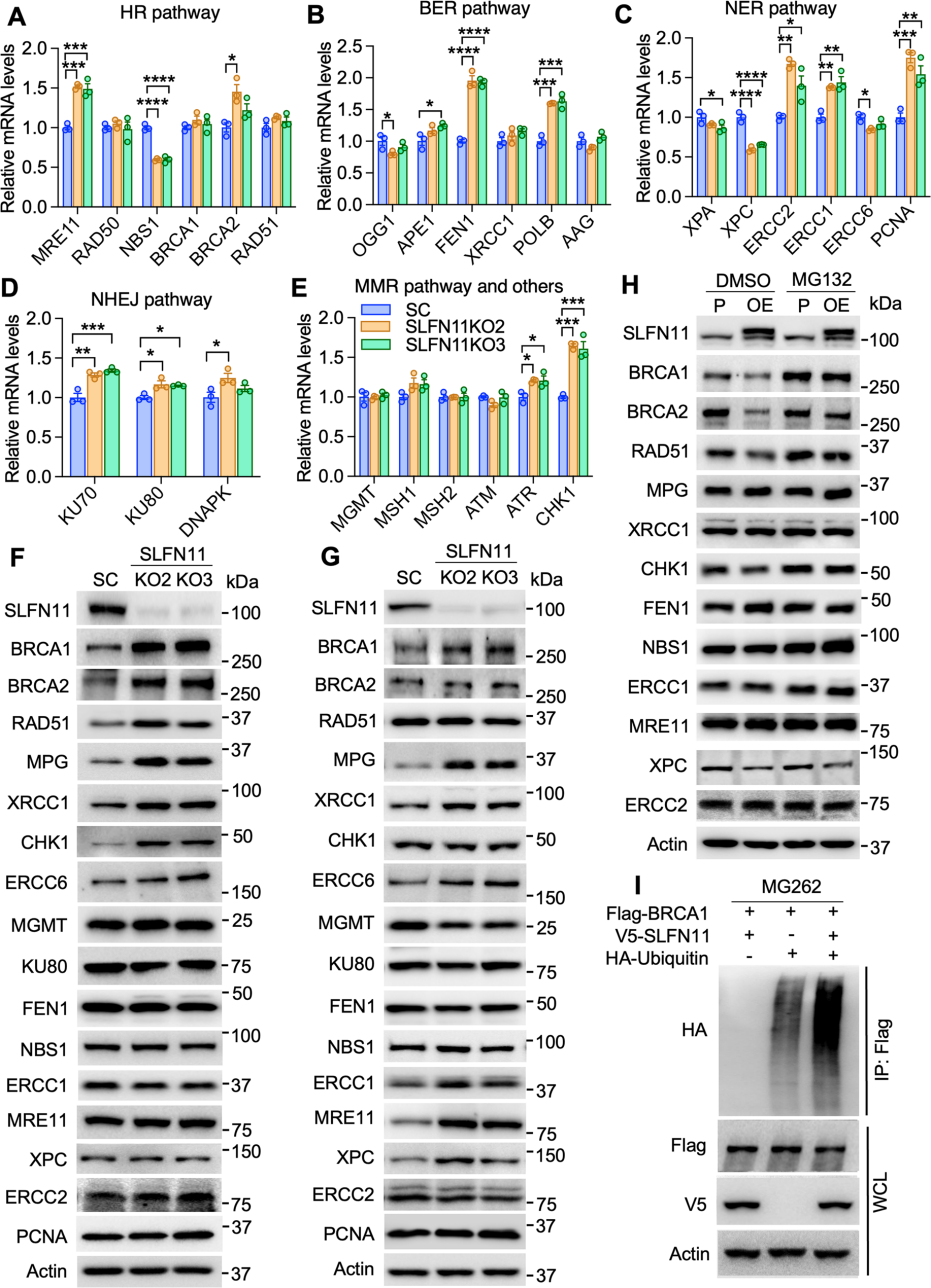
SLFN11 promotes proteasomal degradation of BRCA1 protein. **A**-**E** RT-qPCR analysis of DNA damage repair proteins in SC (blue bar) and SLFN11-KO (yellow & green bars) RCC10 cells (mean ± SEM, *n* = 3). *P < 0.05; **P < 0.01; ***P < 0.001; ****P < 0.0001, by one-way ANOVA with Dunnett’s multiple comparisons test. **F** and **G** Immunoblot analysis of DNA damage repair proteins in SC and SLFN11-KO RCC10 (**F**) and UMRC2 (**G**) cells. **H** Immunoblot analysis of DNA damage repair proteins in parental (P) and SLFN11-overexpressing (OE) RCC10 cells treated with DMSO or MG132 (10 µM) for 6 h. **I** HEK293T cells were co-transfected with Flag-BRCA1, V5-SLFN11, and HA-ubiquitin plasmids and treated with MG262 (5 µM) for 2 h. Whole cell lysates (WCL) were immunoprecipitated with anti-Flag antibody followed by immunoblot analysis with anti-HA antibody

We next sought to dissect the mechanism by which SLFN11 promotes cell death in response to TMZ + Olaparib. To this end, the expression of key proteins in DNA repair pathways in parental and SLFN11-KO2 or KO3 RCC10 cells was analyzed by qRT-PCR and immunoblot assays. SLFN11 KO2 or KO3 had no or little effect on the mRNA expression of most key genes involved in various DNA repair pathways, including HR, BER, NER, NHEJ, and mismatch repair (MMR) (Fig. 5A-E). Modest effects (< 2-fold) were observed for a few genes including MRE11, FEN1, POLB, PCNA and CHK1 at their mRNA but not protein levels (Fig. 5A-G). Interestingly, loss of SLFN11 significantly increased protein levels of BRCA1, BRCA2, RAD51, MPG, XRCC1, and CHK1, but not other DNA repair proteins in RCC10 cells (Fig. 5F, Fig. S6). BRCA1, XRCC1, ERCC6, MRE11, MPG, XPC, and ERCC1 proteins were induced in UMRC2 cells following SLFN11 KO (Fig. 5G, Fig. S7). MGMT protein was downregulated by SLFN11 KO in UMRC2 cells (Fig. 5G, Fig. S7I). We next conducted SLFN11 gain-of-function studies and found that overexpression (OE) of SLFN11 reduced protein levels of BRCA1, BRCA2, and RAD51 only in RCC10 cells (Fig. 5H, Fig. S8). Taken together, these results indicate that SLFN11 consistently regulates BRCA1 expression at the protein level in ccRCC cells, as demonstrated by both gain- and loss-of-function approaches in two independent cell lines.

Interestingly, treatment with a proteasome inhibitor MG132 blocked SLFN11-induced BRCA1 downregulation in RCC10 cells (Fig. 5H, Fig. S8). To determine whether SLFN11 induces BRCA1 protein degradation through the ubiquitin-proteasome system, HEK293T cells were cotransfected with Flag-BRCA1, V5-SLFN11, HA-ubiquitin or empty vector and treated with a proteasome inhibitor MG262 (5 µM) for 2 h. Ubiquitylation assay showed that SLFN11 OE increased BRCA1 ubiquitination in HEK293T cells (Fig. 5I). These findings indicate that SLFN11 enhances ubiquitination and degradation of BRCA1 protein in ccRCC cells.

### SLFN11 attenuates HR activity in response to TMZ and Olaparib combination in ccRCC cells

**Fig. 6:**
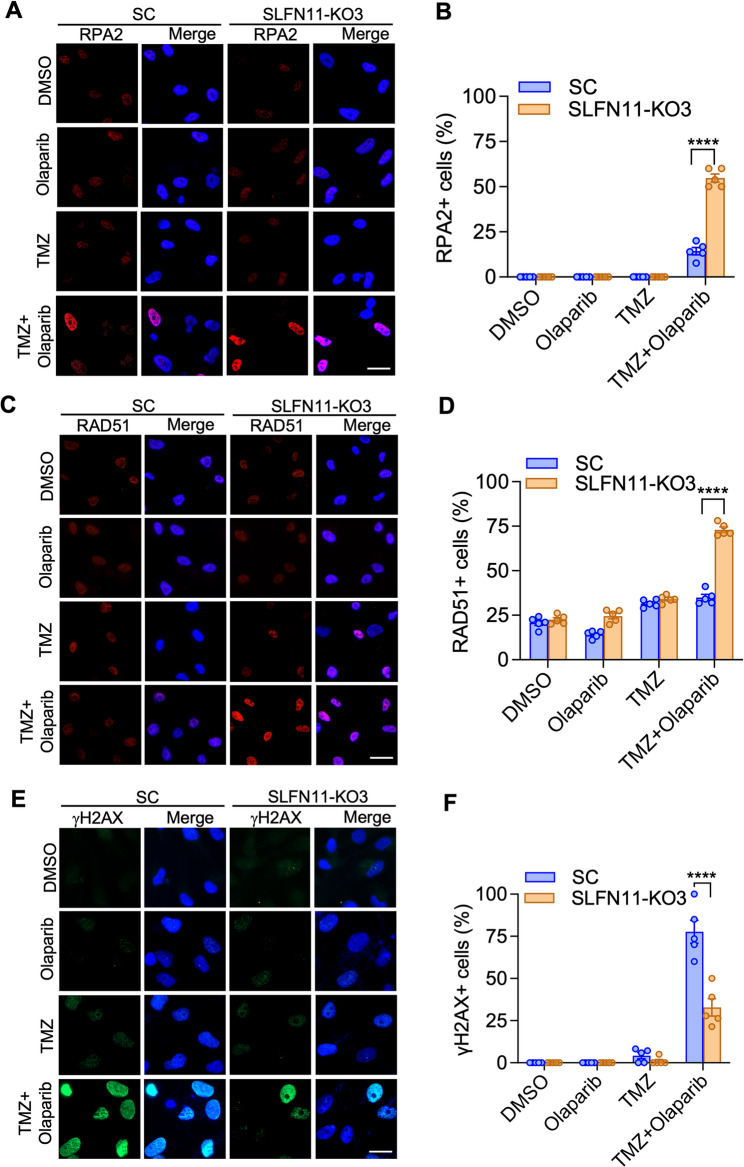
Loss of SLFN11 enhances HR activity in ccRCC cells following TMZ and Olaparib combination treatment. **A** and **B** Representative RPA2 immunostaining images in SC and SLFN11-KO3 RCC10 cells 2 h after treatment with TMZ (500 µM) and/or Olaparib (10 µM) (**A**). The percentage of RPA2-positive cells is quantified in **B** (mean ± SEM, *n* = 5). ****P < 0.0001 by two-way ANOVA with Tukey’s multiple comparisons test. Scale bar, 20 μm. **C** and **D** Representative RAD51 immunostaining images in SC and SLFN11-KO3 RCC10 cells 2 h after treatment with TMZ (500 µM) and/or Olaparib (10 µM) (**C**). The percentage of RAD51-positive cells is quantified in **D** (mean ± SEM, *n* = 5). ****P < 0.0001 by two-way ANOVA with Tukey’s multiple comparisons test. Scale bar, 20 μm. **E** and **F** Representative γH2AX immunostaining images in SC and SLFN11-KO3 RCC10 cells 6 h after treatment with TMZ (500 µM) and/or Olaparib (10 µM) (**E**). The percentage of γH2AX-positive cells is quantified in **F** (mean ± SEM, *n* = 5). ****P < 0.001 by two-way ANOVA with Tukey’s multiple comparisons test. Scale bar, 20 μm

Next, we studied whether SLFN11 regulates HR activity in ccRCC cells following TMZ and/or Olaparib treatment. Parental and SLFN11-KO RCC10 cells were treated with DMSO, TMZ (0.5 mM), Olaparib (10 µM), or TMZ + Olaparib for 2 h. Single treatment with TMZ or Olaparib did not induce RPA2 foci formation in both parental and SLFN11-KO RCC10 cells (Fig. 6A and B). However, RPA2 foci were detected in RCC10 cells following TMZ + Olaparib combination treatment, which were increased by SLFN11 KO (Fig. 6A and B). Similarly, SLFN11 KO significantly increased RAD51 foci formation in RCC10 cells 2 h after TMZ + Olaparib combination treatment (Fig. 6C and D). Consistently, γH2AX foci were decreased in SLFN11-KO RCC10 cells 6 h after TMZ + Olaparib combination treatment, compared with parental RCC10 cells (Fig. 6E and F). These findings indicate that SLFN11 impairs HR activity in response to TMZ + Olaparib in ccRCC cells.

### SLFN11 governs the in vivo sensitivity of ccRCC to TMZ and Olaparib combination therapy

**Fig. 7:**
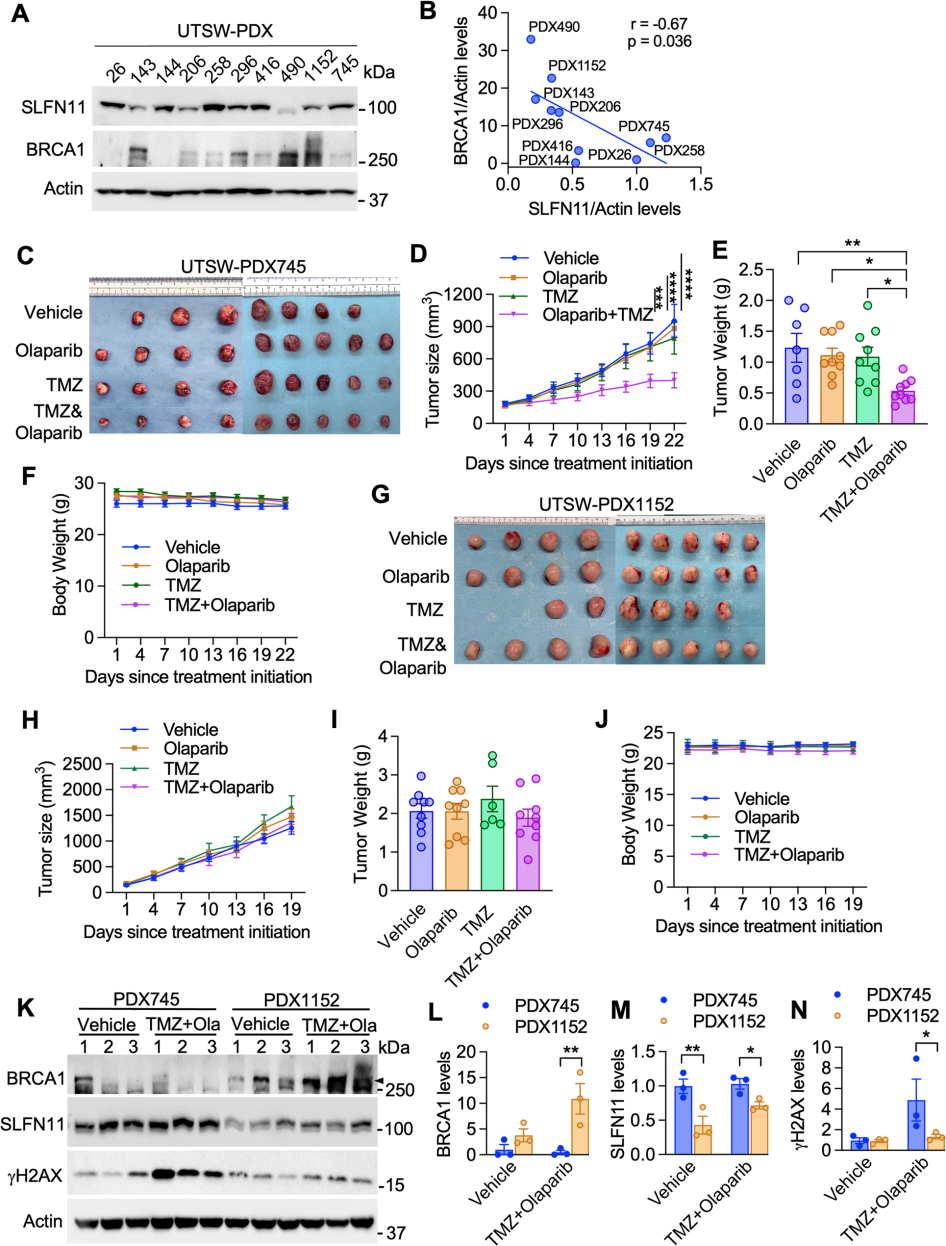
SLFN11 predicts the efficacy of TMZ and Olaparib combination therapy in vivo. **A** Immunoblot analysis of SLFN11 and BRCA1 proteins in human ccRCC PDX tumors. **B** The expression correlation between SLFN11 and BRCA1 in human ccRCC PDX tumors. **C**-**F** UTSW-PDX745 tumors were subcutaneously implanted into male NSG mice and mice were administered Olaparib (50 mg/kg) and/or TMZ (5 mg/kg) for 15 days, with 2 days of rest after every 5 consecutive treatment days (regimen shown in Fig. 2A). Tumor images from two independent studies (**C**), tumor volume (**D**), tumor weight (**E**) and mouse body weights after treatments (**F**) are shown (mean ± SEM, *n* = 7–9). *P < 0.05; **P < 0.01; ***P < 0.001; ****P < 0.0001, by two-way ANOVA Tukey’s multiple comparisons test (**D**) or one-way ANOVA with Dunnett’s multiple comparisons test (**E**). **G**-**J** UTSW-PDX1152 tumors were subcutaneously implanted into male NSG mice and mice were administered Olaparib (50 mg/kg) and/or TMZ (5 mg/kg) for 15 days, with 2 days of rest after every 5 consecutive treatment days (regimen shown in Fig. 2A). Tumor images from two independent studies (**G**), tumor volume (**H**), tumor weight (**I**) and mouse body weights after treatments (**J**) are shown (mean ± SEM, *n* = 6–9). **K**-**N** BRCA1, SLFN11 and γH2AX levels and quantification in UTSW-PDX745 and UTSW-PDX1152 tumors treated with vehicle or TMZ + Olaparib

We next assessed the expression correlation between SLFN11 and BRCA1 proteins in human ccRCC PDX tumors, which have been known to closely recapitulate the characteristics of human ccRCC [33]. ccRCC PDX tumors with higher levels of SLFN11 protein, including UTSW-PDX26, UTSW-PDX144, UTSW-PDX258, UTSW-PDX296, UTSW-PDX416, and UTSW-PDX745, expressed lower levels of BRCA1 protein, as compared to other 4 PDX tumors (Fig. 7A). In these PDX tumors, SLFN11 protein levels showed a significant inverse correlation with BRCA1 protein levels (*r* = − 0.67, *p* = 0.036) (Fig. 7B). To determine a role of SLFN11 in regulating the sensitivity of ccRCC to TMZ + Olaparib combination treatment in vivo, we compared the killing effect of TMZ + Olaparib in SLFN11-high (UTSW-PDX745) and -low (UTSW-PDX1152) ccRCC PDX mouse models. UTSW-PDX745 or UTSW-PDX1152 tumors were subcutaneously implanted into the flank of male NSG mice. Once tumor volume reached ~ 100 mm³, mice were administered Olaparib (50 mg/kg), TMZ (5 mg/kg), TMZ + Olaparib, or vehicle daily for five continuous days, followed by two-day break, for a total of three weeks. The combination treatment with TMZ and Olaparib significantly reduced UTSW-PDX745 tumor growth, whereas their single treatment failed to do so (Fig. 7C-E). The body weight was not altered by these treatments in mice bearing UTSW-PDX745 tumors (Fig. 7F). In contrast, UTSW-PDX1152 tumors were resistant to treatment with either TMZ, Olaparib, or a combination of TMZ + Olaparib (Fig. 7G-J). The expression of SLFN11 and BRCA1 was validated in UTSW-PDX745 and UTSW-PDX1152 tumors treated with vehicle or TMZ + Olaparib. As expected, UTSW-PDX745 tumors expressed higher SLFN11 and lower BRCA1 levels, whereas UTSW-PDX1152 tumors showed the opposite pattern, regardless of drug treatment (Fig. 7K-M). Consistent with these expression profiles and distinct treatment response, TMZ + Olaparib induced significantly higher DNA damage in UTSW-PDX745 than in UTSW-PDX1152 (Fig. 7K, N). These findings indicate that SLFN11 may be a biomarker that predicts a response to TMZ + Olaparib combination treatment in ccRCC.

Consistent with our findings that Olaparib or TMZ monotherapy showed little to moderate ccRCC growth inhibition in vitro and in vivo (Figs. 1, 2 and 7), CellMinerCDB analysis of the GDSC-MGH-Sanger datasets showed a moderate correlation between SLFN11 and TMZ sensitivity in human kidney cancers (*r* = 0.39, *p* = 0.026) and no correlation between SLFN11 and Olaparib sensitivity (*r* = 0.08, *p* = 0.62) (Fig. S9A and B). Pan-cancer analyses similarly revealed only weak associations for SLFN11-TMZ (*r* = 0.24, *p* = 1.4 × 10⁻¹³) and SLFN11-Olaparib (*r* = 0.27, *p* = 7.4 × 10⁻¹⁶) across all human cancers in the GDSC-MGH-Sanger datasets (Fig. S9C and D). Collectively, these findings highlight that SLFN11 is insufficient to predict sensitivity to TMZ or Olaparib monotherapy, reinforcing that an SLFN11-dependent therapeutic vulnerability emerges only under specific combination treatment contexts.

## Discussion

In the present study, we demonstrated the lethal effect of a subgroup of alkylating agents when combined with PARP inhibitors in ccRCC cells in vitro and in PDX tumor models in vivo. PARP inhibitors are currently used in cancer treatment due to their synthetic lethality mechanism. Mutant BRCA1/2 were the first identified synthetic lethal partners of PARP inhibitors [2]. Since then, the concept of PARP inhibitor-mediated synthetic lethality has evolved, extending the use of PARP inhibitors to target cancer cells with BRCAness [9]. Our present studies demonstrated that alkylating agent treatment activates a PARP-1 dependency in SLFN11-high ccRCC cells, which triggers PARP-1-dependent DNA repair to resolve DNA damage for survival. As such, SLFN11-high ccRCC cells are vulnerable to PARP inhibitor upon alkylating agent treatment, but not to either agent alone (Figs. 1, 2 and 7). These findings uncover a rational therapeutic strategy that can sensitize tumors to PARP inhibitors and expand potential application of PARP inhibitors for the treatment of a subtype of BRCA1/2-WT cancers.

PARP-1 functions are highly context-dependent. We and others have shown that PARP-1 has a dual role in cell survival and death [20]. Upon mild DNA damage, PARP-1 recruits DNA repair proteins and facilitates DNA repair, thereby contributing to cell survival. However, in response to severe DNA damage, PARP-1 activation elicits a death signal to induce genomic DNA fragmentation leading to cell death, also known as PARP-1-dependent cell death (PARthanatos) [32]. Accordingly, the opposite effects of PARP-1 loss in ccRCC cells versus breast cancer MDA-MB-231 cells in response to the same methylating agent (MNNG; Fig. S2) likely reflect differences in how these cell types process methylating DNA lesions and route PARP-1 signaling toward repair/survival versus PARthanatos. In contrast to its DNA repair function in ccRCC, PARP-1 enhances alkylating agent-induced cell death in breast cancer, which can be blocked by its genetic or pharmacological inhibition (Fig. S2). Thus, the dual functions of PARP-1 in mediating cell survival and death are well demonstrated by our observations in ccRCC and breast cancer cells, respectively. As a result, combined treatment with PARP-1 inhibitor and alkylating agent kills ccRCC cells but not breast cancer cells. These cell type-specific outcomes may reflect differences in SLFN11/HR proficiency, BER/replication-stress responses, and PAR turnover and cellular NAD^+^ metabolism. Interestingly, PARP-1 activity is much higher in ccRCC cells than breast cancer cells, although PARP-1 protein levels are comparable in these cell lines. NAD^+^ is a crucial substrate for PARP-1 activation. The extent of PARP activation is also regulated by the dePARylase poly(ADP-ribose) glycohydrolase (PARG). Further studies are needed to investigate the roles of NAD^+^ and PARG in PARP-1 hyperactivation in ccRCC cells. Nevertheless, our study suggests a cell-type-specific role of PARP-1 in cell survival and death.

We showed that the lethal effect of alkylating agent and PARP inhibitor combination on ccRCC cells is VHL-independent, although the VHL-HIF signaling is a hallmark in ccRCC and plays a critical role in tumor progression [36]. Instead, we here identified SLFN11 as a key factor that determines the efficacy of alkylating agent and PARP inhibitor combination in ccRCC. The levels of SLFN11 are much higher in ccRCC cells than breast cancer cells, consistent with findings from human tumors in the TCGA cohorts. Previous studies have revealed a close link between SLFN11 and DNA damage response [30]. We found that SLFN11 loss increases HR activity in ccRCC cells, which aligns with a previous study [30]. SLFN11 expression correlates with the efficacy of PARP inhibitors in small cell lung cancer [26]. Our findings support the notion that SLFN11 is a biomarker that predicts the efficacy of the combination treatment with DNA damage agents and PARP-1 inhibitors in cancer cells. We also showed specific loss of ERCC6 in ccRCC cells. Future investigations are needed to understand the role of ERCC6 in alkylating agent and PARP inhibitor combination-induced ccRCC cell death.

**Fig. 8:**
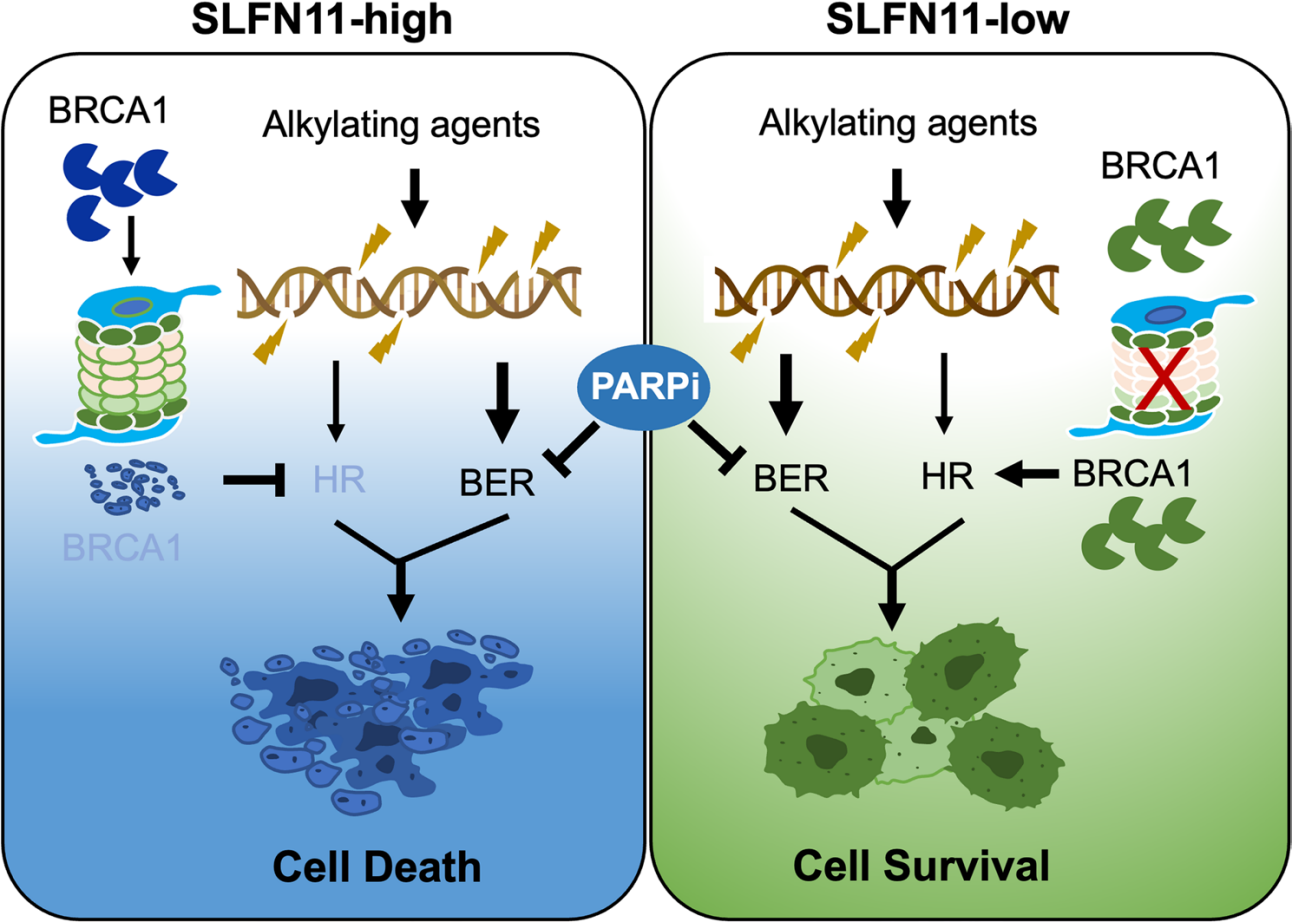
The proposed mechanism underlying alkylating agent and PARP inhibitor combination -induced ccRCC cell death. High levels of SLFN11 enhances proteasomal degradation of BRCA1 protein in ccRCC cells, leading to reduced HR activity. As such, ccRCC cells utilize BER to repair DNA damage upon treatment with alkylating agent, which renders cancer cells vulnerable to PARP inhibitor

SLFN11 was shown to inhibit ATR and ATM translation through cleavage of type II tRNAs, a mechanism critical for DNA damage-induced cell death [29]. However, SLFN11 does not alter ATR, ATM or CHK1 at either mRNA or protein levels in ccRCC cells. Instead, we found that SLFN11 regulates BRCA1 protein stability in ccRCC cells without affecting BRCA1 mRNA levels. SLFN11 may also modulate BRCA2 in a manner similar to BRCA1 in certain ccRCC cells. Several E3 ubiquitin ligases, including HERC2, HUWE1, and FBXO44, have been shown to target BRCA1 protein for degradation [37–39]. While the E3 ubiquitin ligase responsible for SLFN11-induced BRCA1 protein degradation remains to be identified in ccRCC, previous reports have shown that SLFN11 interacts with CUL4 E3 ubiquitin ligase [40]. Nevertheless, our findings, along with others, suggest that while SLFN11 is a key regulator of DNA damage response proteins at their protein levels to influence cancer cell vulnerability to DNA damage agents (Fig. 8), the specific pathways mediating drug sensitivity are likely cancer type-dependent.

Chemotherapy is not a current standard of care in patients with ccRCC because ccRCC has been traditionally considered resistant to chemotherapy. In line with this notion, we found that the individual treatment with alkylating agents, although used clinically as chemotherapeutics, shows little efficacy in ccRCC in vitro and in mice. However, selective alkylating agents induce hyperactivation of PARP-1 in ccRCC cells, which renders tumors vulnerable to PARP-1 inhibitors. Notably, among chemical agents we tested, the DNA-methylating alkylators TMZ and STZ generate sustained PARP-1 activation. Their methyl-adduct profiles are preferentially repaired through MGMT and the BER pathway, producing single-strand break intermediates that strongly engage PARP-1 and thereby create a PARP-1-dependent state that synergizes with PARP inhibition. In contrast, although other alkylators can trigger PARP-1 activation, they may not generate the same lesion-specific BER intermediates and thus fail to induce a comparable PARP-1 dependency in ccRCC. Our chemical screen, together with comparative analyses of published studies in other cancer types, suggests that PARP-1-dependent drug responses are highly cell type- and context-dependent, reflecting biologically meaningful variation across tumors. Recent clinical trials have shown potential clinical benefits of TMZ and Olaparib or Rucaparib combination in small cell lung cancer or melanoma [41, 42]. Our study reveals that BRCA1/2-WT ccRCC harbors an unexpected, targetable vulnerability to alkylating agent & PARP inhibitor combination therapy, illustrating how the lesion class, repair routing, and tumor-specific DNA repair wiring together determine therapeutic responsiveness. SLFN11 and BRCA1 therefore represent rational, complementary candidate biomarkers for stratifying ccRCC patients for TMZ and PARP inhibitor combination therapy. However, our data suggest that SLFN11/BRCA1 status may be necessary but not sufficient, because baseline PARP-1 activity may further modulate sensitivity to the combination.

## Conclusions

Our work identifies the combination of TMZ or STZ with PARP inhibitor as an effective treatment strategy for ccRCC tumors with high SLFN11 expression and elevated PARP-1 activity. These approaches have a rapid translational potential, considering that all compounds have been approved by FDA for cancer treatment. Although our present data indicate that SLFN11 predicts sensitivity to TMZ and PARP inhibitor combination therapy in kidney cancer, this relationship may not generalize across all tumor types but may extend to select additional contexts, underscoring tissue- and cell type-specific nature of SLFN11-dependent drug responses.

## Supplementary Information

Supplementary Material 1.

## Data Availability

All datasets used/analyzed during the current study have been included in this article and its supplementary materials.

## Abbreviations

5-FU: 5-Fluorouracil
ccRCC: Clear cell renal cell carcinoma
DPQ: 3,4-dihydro-5-[4-(1-piperidinyl)butoxy]-1(2H)-isoquinolinone
ENU: N-ethyl-N-nitrosourea
HR: Homologous recombination
KO: Knockout
MGMT: O6-methylguanine-DNA methyltransferase
MMS: Methyl methanesulfonate
MNNG: N-methyl-N′-nitro-N-nitrosoguanidine
NHEJ: Non-homologous end joining
NSG: NOD-SCID-IL2RGKO
OE: Overexpression
PAR: Poly(ADP-ribose)
PARP: Poly(ADP-ribose) polymerase
PDX: Patient-derived xenograft
PI: Propidium iodide
qRT-PCR: Quantitative reverse transcription-polymerase chain reaction
SC: Scrambled control
SLFN11: Schlafen family member 11
STZ: Streptozocin
TMZ: Temozolomide
VHL: Von Hippel–Lindau
WT: Wild type
XRCC1: X-ray repair cross-complementing protein 1
