# Supplementary material for: Alkylating agents activate an SLFN11-dependent vulnerability that confers PARP-1 inhibitor sensitivity in kidney cancer
Source: J Exp Clin Cancer Res. 2026 Jun 9;45:176. doi: 10.1186/s13046-026-03743-1 (PMC13471403; doi:10.1186/s13046-026-03743-1)
Supplement: Supplementary file 1 [file 13046_2026_3743_MOESM1_ESM.pdf]

Supplementary information

Alkylating agents activate an SLFN11-dependent vulnerability that confers PARP-1 inhibitor sensitivity in kidney cancer

Chenliang Wang[1], Ming Zhu[1], Lei Bao[1], Vanina Toffessi Tcheuyap[2,3], James Brugarolas[2,3], Weibo Luo[1,4,*], Yingfei Wang[1,5,6,*]

[1]Department of Pathology, UT Southwestern Medical Center, Dallas, TX 75390, USA.
[2]Kidney Cancer Program, Harold C. Simmons Comprehensive Cancer Center, UT Southwestern Medical Center, Dallas, TX 75390, USA.
[3]Department of Internal Medicine, UT Southwestern Medical Center, Dallas, TX 75390, USA.
[4]Department of Pharmacology, UT Southwestern Medical Center, Dallas, TX 75390, USA.
[5]Department of Neurology, UT Southwestern Medical Center, Dallas, TX 75390, USA.
[6]Peter O'Donnell Jr. Brain Institute, UT Southwestern Medical Center, Dallas, TX 75390, USA.

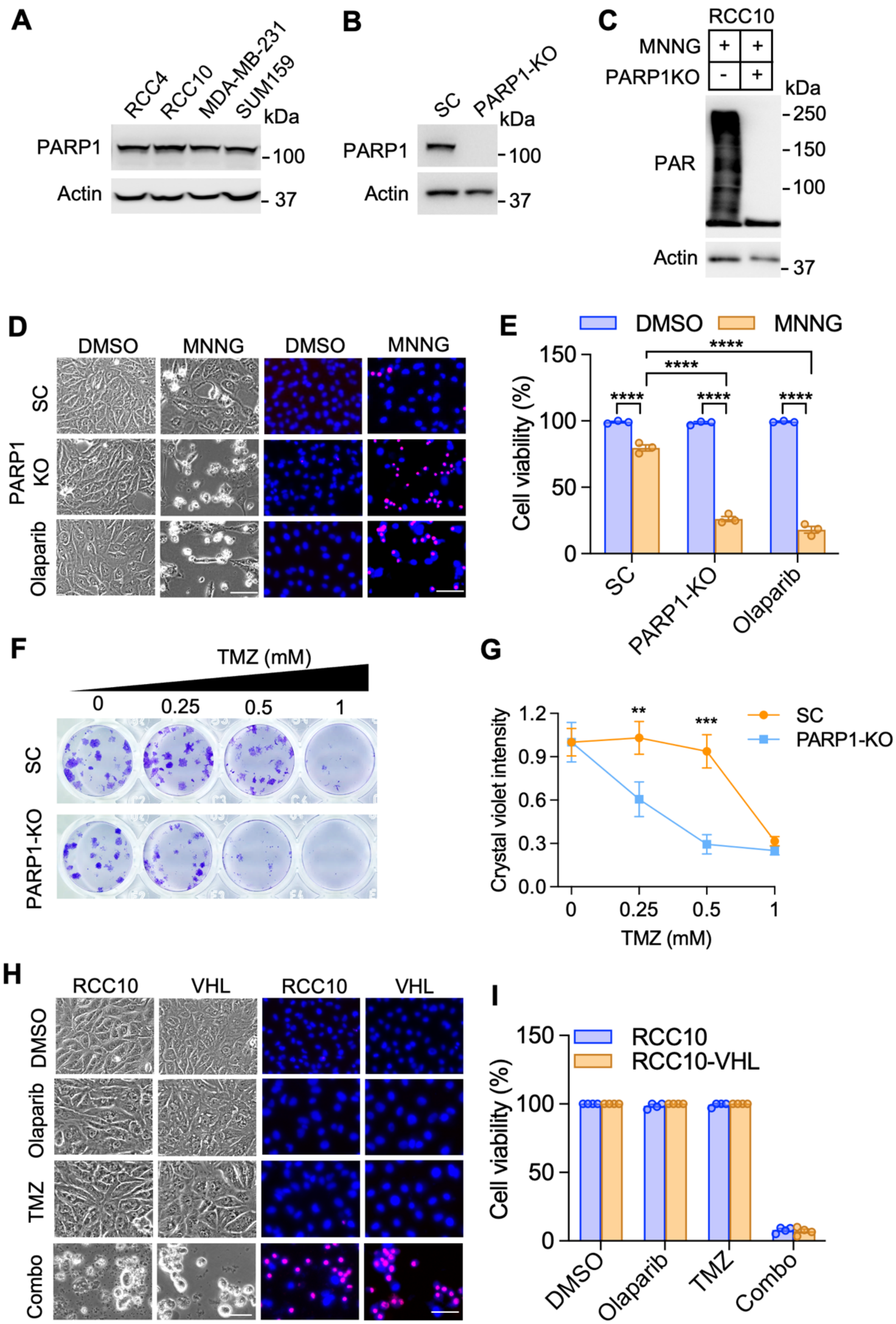

Supplementary Figure 1. Alkylating agents render ccRCC cells sensitive to PARP inhibition.

(A) Immunoblot analysis of PARP1 in various cancer cells. (B) Immunoblot analysis of PARP1 in scrambled control (SC) and PARP1-KO RCC10 cells. (C) Immunoblot analysis of PARP-1 activation in parental and PARP1-KO RCC10 cells 15 min after treatment with MNNG (50 μM). (D and E) Representative cell death images in SC and PARP1-KO RCC10 cells treated with vehicle or MNNG (30 μM) and in RCC10 cells treated with MNNG (30 μM) and/or Olaparib (10 μM) (D). The percentage of PI-positive cells (red) is quantified in E (mean ± SEM, n = 3-4). ****$P < 0.0001$ by two-way ANOVA with Tukey's multiple comparisons test. Scale bar, 100 μm. (F and G) Representative colony survival in SC and PARP1-KO RCC10 cells treated with vehicle or TMZ for 10 days (F). The intensity of crystal violet is quantified in G (mean ± SEM, n = 3). **$P < 0.01$; ***$P < 0.001$ by two-way ANOVA with Tukey's multiple comparisons test. (H and I) Representative cell death images in RCC10 and RCC10-VHL cells treated with TMZ (500 μM) and/or Olaparib (10 μM) for 10 days (H). The percentage of PI-positive cells (red) is quantified in I (mean ± SEM, n = 4). Scale bar, 100 μm.

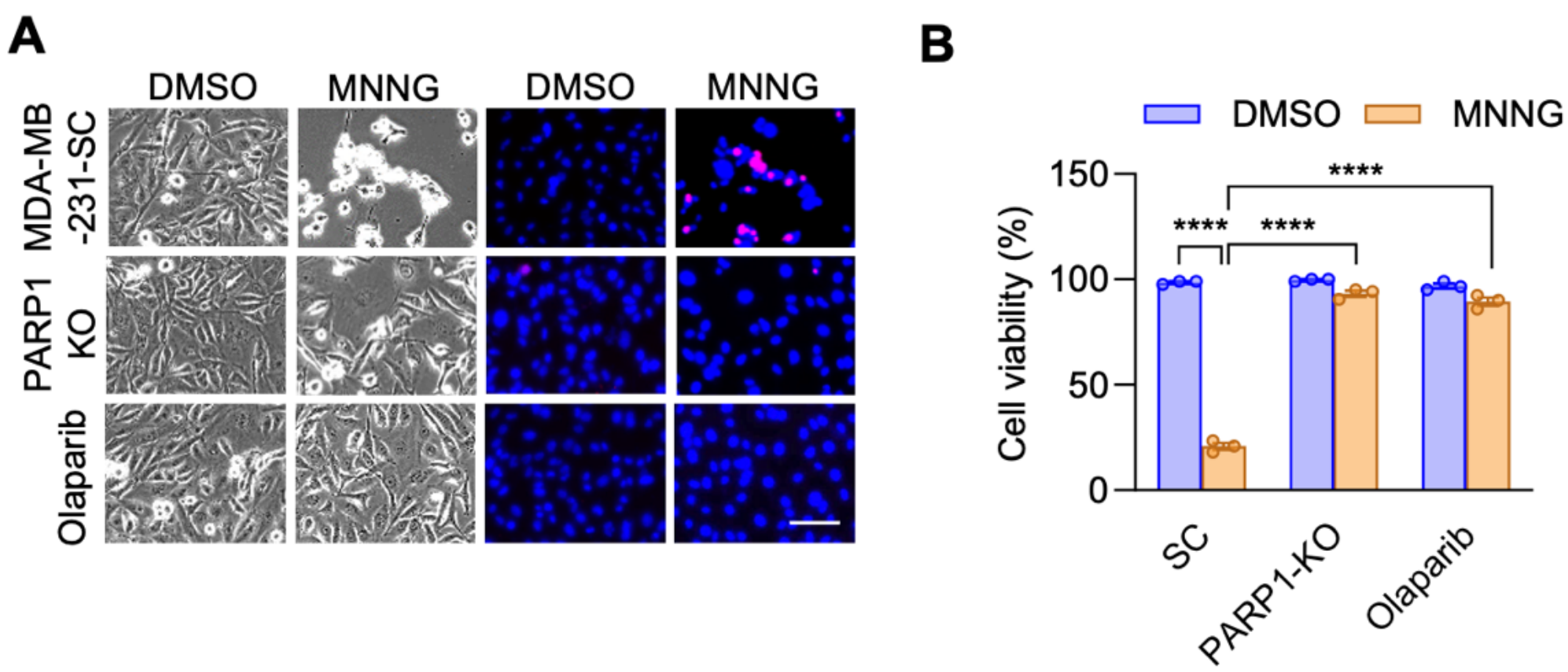

Supplementary Figure 2. Loss of PARP1 protects breast cancer cells against alkylating agent-induced cell death.

(A and B) Representative cell death images in SC and PARP1-KO MDA-MB-231 cells treated with vehicle or MNNG (50 μM) and MDA-MB-231 cells treated with MNNG (50 μM) and/or Olaparib (10 μM) (A). The percentage of PI-positive cells (red) is quantified in B (mean ± SEM, $n = 3$). ****$P < 0.0001$ by two-way ANOVA with Tukey's multiple comparisons test. Scale bar, 100 μm.

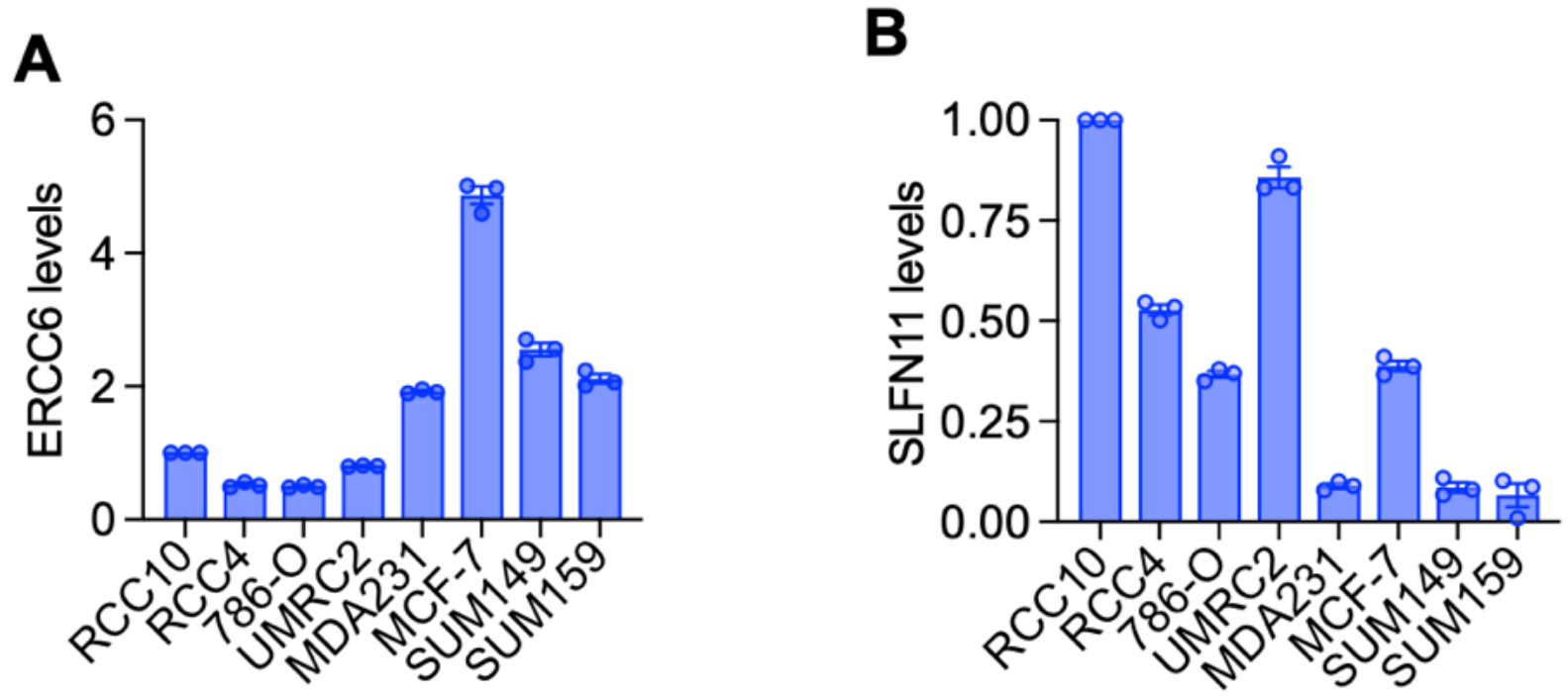

Supplementary Figure 3. Expression of ERCC6 and SLFN11 proteins in various cancer cell lines.

(A and B) Quantification of ERCC6 (A) and SLFN11 (B) protein expression in kidney and breast cancer cell lines (mean ± SEM, n = 3).

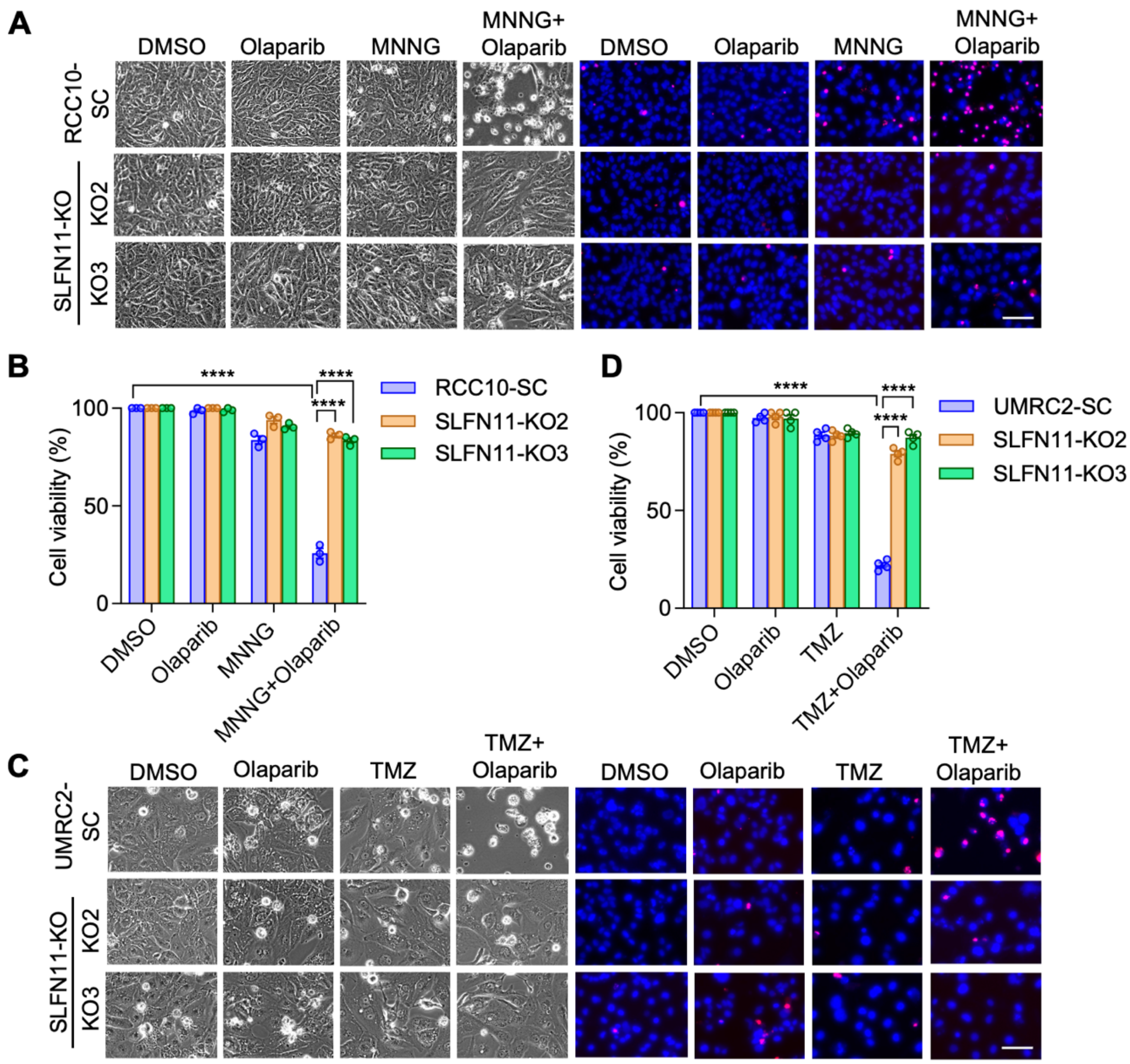

Supplementary Figure 4. Loss of SLFN11 protects ccRCC cells against an alkylating agent and PARP inhibitor combination treatment.

(A and B) Representative cell death images in SC and SLFN11-KO RCC10 cells 24 h after treatment with MNNG (30 µM) and/or Olaparib (10 µM) (A). The percentage of PI-positive cells (red) is quantified in B (mean ± SEM, n = 3). Scale bar, 100 µm. ****P < 0.0001 by two-way ANOVA Tukey's multiple comparisons test. (C and D) Representative cell death images in SC and SLFN11-KO UMRC2 cells 24 h after treatment with TMZ (500 µM) and/or Olaparib (10 µM) (D). The percentage of PI-positive cells (red) is quantified in C (mean ± SEM, n = 4). Scale bar, 100 µm. ****P < 0.0001 by two-way ANOVA with Tukey's multiple comparisons test.

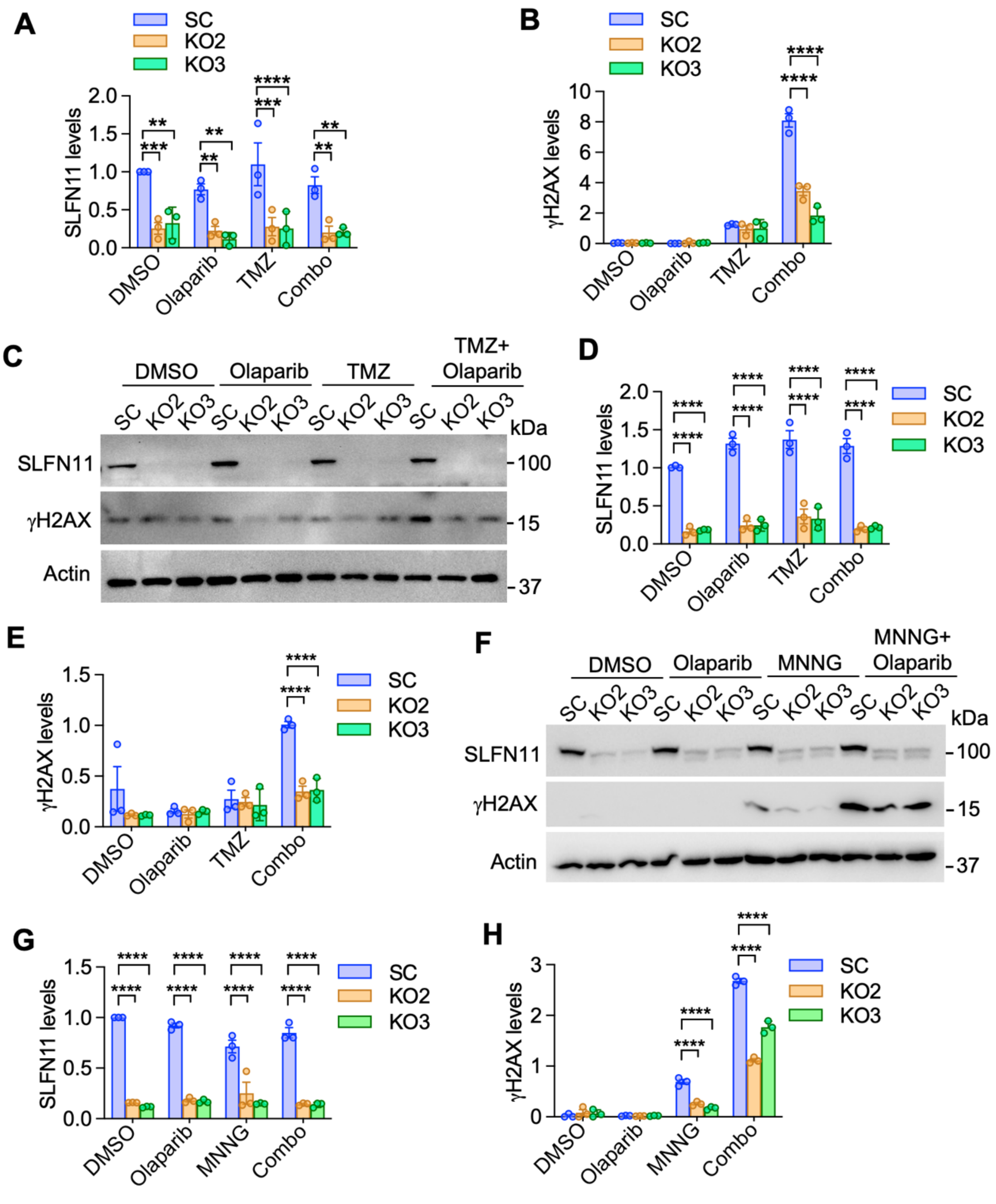

Supplementary Figure 5. SLFN11 loss blocks DNA damage induced by TMZ&Olaparib combo treatment in ccRCC cells.

(A and B) Quantification of SLFN11 (A) and γH2AX (B) expression in SC and SLFN11-KO RCC10 cells treated with TMZ (500 μM) and/or Olaparib (10 μM) for 24 h (mean ± SEM, $n = 3$). ***P* < 0.01, ****P* < 0.001, *****P* < 0.0001 by two-way ANOVA with Tukey's multiple comparisons test. (C-E) Immunoblot analysis of SLFN11 and γH2AX in SC and SLFN11-KO UMRC2 cells treated with TMZ (500 μM) and/or Olaparib (10 μM) for 24 h (C). The SLFN11 (D) and γH2AX (E) band intensity is quantified (mean ± SEM, $n = 3$). *****P* < 0.0001 by two-way

ANOVA with Tukey's multiple comparisons test. (F-H) Immunoblot analysis of SLFN11 and γH2AX in SC and SLFN11-KO RCC10 cells treated with MNNG (30 μM) and/or Olaparib (10 μM) for 24 h (F). The SLFN11 (G) and γH2AX (H) band intensity is quantified (mean ± SEM, $n = 3$). ****$P < 0.0001$ by two-way ANOVA with Tukey's multiple comparisons test.

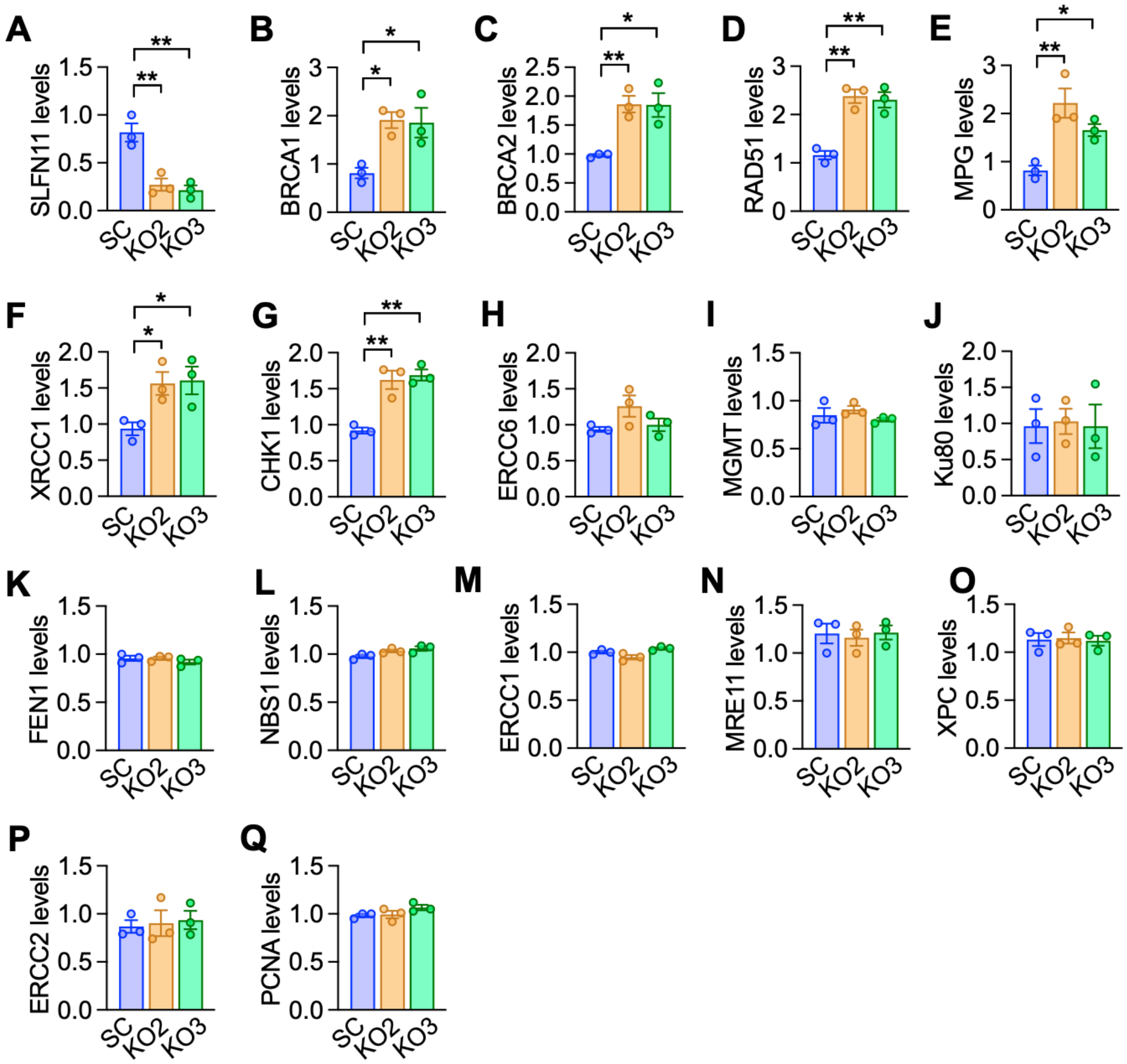

Supplementary Figure 6. Effects of SLFN11 loss on the expression of DNA damage repair proteins in ccRCC cells.

(A-Q) Quantification of indicated proteins in SC and SLFN11-KO RCC10 cells, with representative images shown in Fig. 5F (mean ± SEM, $n = 3$). $^{*}P < 0.05$, $^{**}P < 0.01$, by one-way ANOVA with Dunnett's multiple comparisons test.

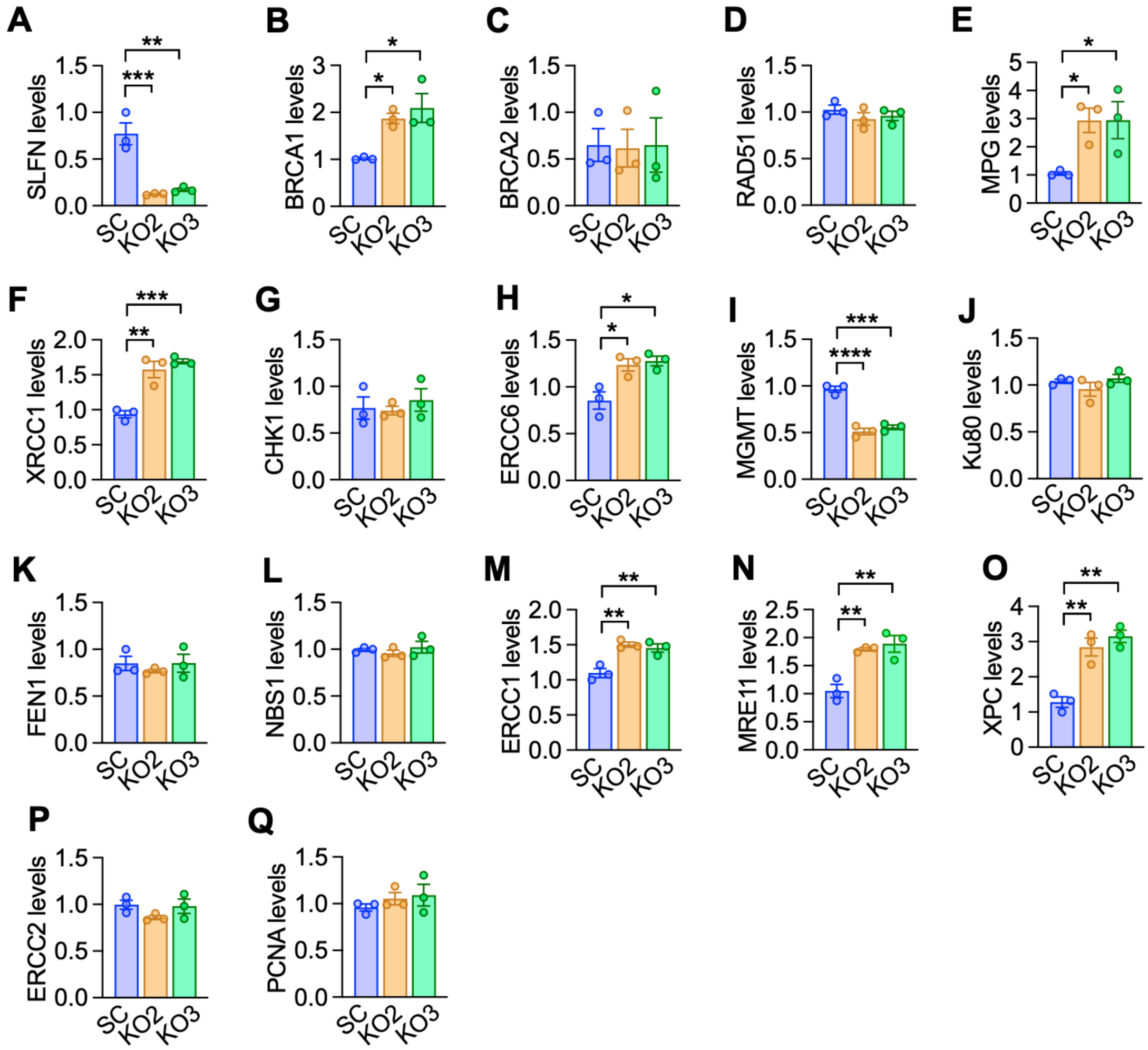

Supplementary Figure 7. Effects of SLFN11 loss on the expression of DNA damage repair proteins in ccRCC cells.

(A-Q) Quantification of indicated proteins in SC and SLFN11-KO UMRC2 cells, with representative images shown in Fig. 5G (mean ± SEM, n = 3). *$P < 0.05$, **$P < 0.01$, ***$P < 0.001$, ****$P < 0.0001$, by one-way ANOVA with Dunnett's multiple comparisons test.

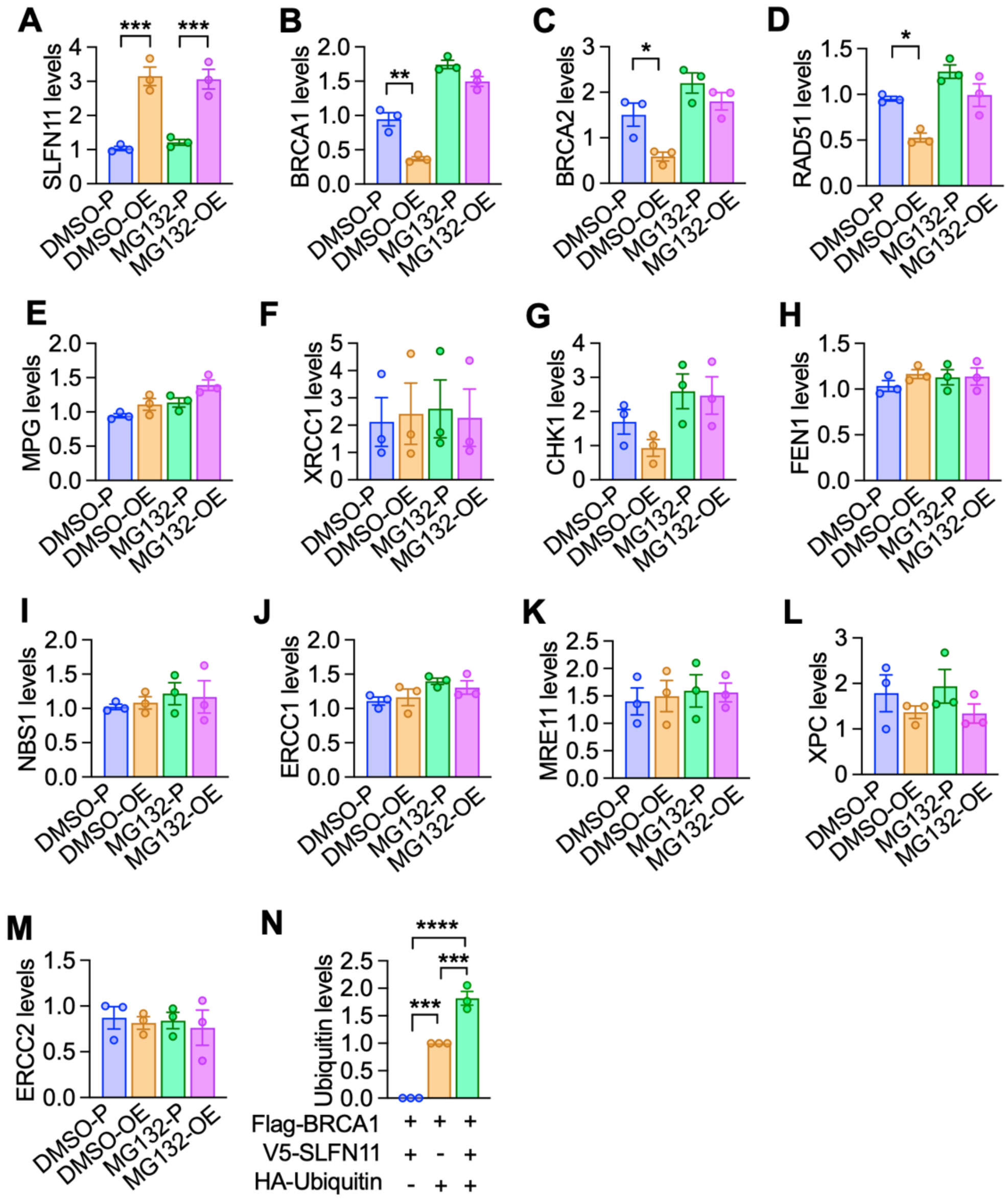

Supplementary Figure 8. Effects of SLFN11 overexpression on the expression of DNA damage repair proteins in ccRCC cells.

(A-M) Quantification of indicated proteins in parental (P) and SLFN11-overexpressed (OE) RCC10 cells treated with DMSO or MG132 (10 μM) for 6 h, with representative images shown in Fig. 5H (mean ± SEM, $n = 3$). *$P < 0.05$, **$P < 0.01$, ***$P < 0.001$, ****$P < 0.0001$, by one-way ANOVA with Tukey's multiple comparisons test. (N). Quantification of ubiquitinated SLFN11 levels in HEK293T cells, with representative images shown in Fig. 5I (mean ± SEM, $n = 3$). ***$P < 0.001$, ****$P < 0.0001$, by one-way ANOVA with Tukey's multiple comparisons test.

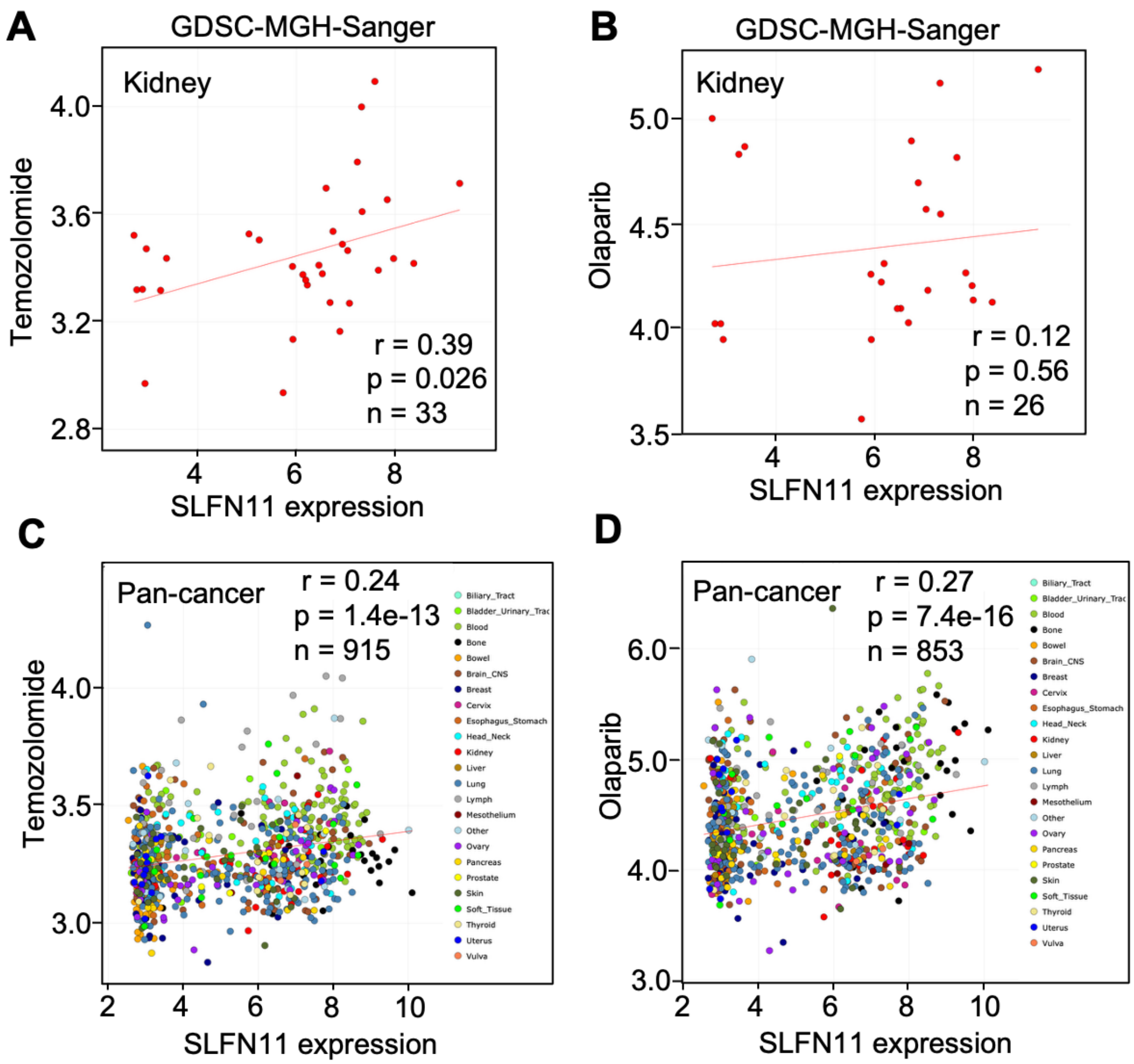

Supplementary Figure 9. CellMinerCDB-based correlation analysis between SLFN11 mRNA levels and TMZ or Olaparib sensitivity across human cancers.

(A) Correlation analysis of SLFN11 expression and TMZ sensitivity in kidney cancer. (B) Correlation analysis of SLFN11 expression and Olaparib sensitivity in kidney cancer. (C) Correlation analysis of SLFN11 expression and TMZ sensitivity in pan human cancers. (D) Correlation analysis of SLFN11 expression and Olaparib sensitivity in pan human cancers. Data were extracted from the GDSC-MGH-Sanger datasets through the CellMinerCDB platform. Pearson correlation coefficients (*r*) were calculated to assess association strength, with $|r| = 0.1$–0.3 considered weak, 0.3–0.5 moderate, and > 0.5 strong.

Supplementary Table 1. The list of drugs/chemicals used in our cell viability screen.

Chemical name	Abbreviation	Class	Dose	Treatment duration
N-methyl-N'-nitro-N-nitrosoguanidine	MNNG	DNA alkylating agent	30 µM	24 h
Methyl methanesulfonate	MMS	DNA alkylating agent	4 mM	24 h
Mechlorethamine		DNA alkylating agent	20 µM	48 h
Carmustine	BCNU	DNA alkylating agent	200 µM	72 h
Temozolomide	TMZ	DNA alkylating agent	1 mM	48 h
Thiotepa	-	DNA alkylating agent	50 µM	48 h
Cisplatin	-	Alkylating-like agent	20 µM	72 h
N-ethyl-N-nitrosourea	ENU	DNA alkylating agent	10 mM	48 h
Cyclophosphamide	Cytoxan	DNA alkylating agent	1 mM	48 h
Busulfan	-	DNA alkylating agent	200 µM	48 h
Streptozocin	STZ	DNA alkylating agent	1 mM	48 h
β-Lapachone	-	Topoisomerase I inhibitor	4 µM	48 h
Camptothecin	CPT	Topoisomerase I inhibitor	500 nM	48 h
Etoposide	-	Topoisomerase II inhibitor	6 µM	48 h
Doxorubicin	DOX	Anthracycline antibiotic; Topoisomerase II inhibitor	400 nM	72 h
Gemcitabine	-	Antimetabolite/Pyrimidine analog	500 nM	48 h
Fluorouracil	5-FU	Antimetabolite/Pyrimidine analog	200 µM	96 h
Paclitaxel	-	Antimicrotubule agent	100 nM	72 h
Mercaptopurine	6-MP	Antimetabolite /Purine analog	200 µM	96 h
6-Thioguanine	6-TG	Antimetabolite /Purine analog	300 µM	96 h
Methotrexate	MTX	Antimetabolite /Folic acid antagonist	400 µM	96 h

Supplementary Table 2. Oligonucleotide sequences of sgRNAs and PCR primers used for plasmid constructs

Gene	Primer sequence
PARP1- sgRNA	Forward: 5'-CACCGCGAGTCGAGTACGCCAAGAG-3'
	Reverse: 5'-AAACCTCTTGGCGTACTCGACTCGC-3'
SLFN11- sgRNA2	Forward: 5'-CACCGTACACTGGTCTGCTAAGGGG-3'
	Reverse: 5'-AAACCCCCTTAGCAGACCAGTGTAC-3'
SLFN11- sgRNA3	Forward: 5'-CACCGTGTCAGCTGAGTCTATCTAG-3'
	Reverse: 5'-AAACCTAGATAGACTCAGCTGACAC-3'
SLFN11(pLVX-Ubc-FLAG-N)	Forward: 5'-TGACGATGACAAGCATATGCTCATGGAGGCAAATCAGTGCC-3'
	Reverse: 5'-AGAATTATCTAGATGTACAGGACTAATGGCCACCCCACG-3'
SLFN11 (pcDNA3.1-V5)	Forward: 5'-CCCAAGCTTATGGAGGCAAATCAGTGCCC-3'
	Reverse: 5'-CCGGATATCCTAATGGCCACCCCACGG-3'

Supplementary Table 3. The list of antibodies used in this study

Antibodies	Source	Identifier
anti-PAR	MilliporeSigma	Cat# MABE1031
anti-Actin	Proteintech	Cat# 66009-1-AP
anti-PARP1	Proteintech	Cat# 22999-1-AP
anti-ERCC6	Proteintech	Cat# 24291-1-AP
anti-γH2AX	MilliporeSigma	Cat# 05-636-AF647
anti-SLFN11	Proteintech	Cat# 26060-1-AP
anti-BRCA1	Proteintech	Cat# 22362-1-AP
anti-RAD51	Proteintech	Cat# 14961-1-AP
anti-MPG	Proteintech	Cat# 11481-2-AP
anti-XRCC1	Proteintech	Cat# 21468-1-AP
anti-CHK1	Santa Cruz Biotechnology	Cat# sc-8408
anti-MGMT	Proteintech	Cat# 67476-1-Ig
anti-KU80	ThermoFisher	Cat# PA5-17454
anti-FEN1	Proteintech	Cat# 14768-1-AP
anti-NBS1	Proteintech	Cat# 55025-1-AP
anti-ERCC1	Proteintech	Cat# 14586-1-AP
anti-MRE11	Proteintech	Cat# 10744-1-AP
anti-XPC	Cell Signaling Technology	Cat# 12701T
anti-ERCC2	Proteintech	Cat# 10818-1-AP
anti-BRCA2	Proteintech	Cat# 19791-1-AP
anti-HA	MilliporeSigma	Cat# H9658
anti-V5	ThermoFisher	Cat# R96025
anti-FLAG	MilliporeSigma	Cat# F3165
anti-RPA32/RPA2	Fortis Life Sciences	Cat# 50-155-7197
anti-PARP2	Proteintech	Cat# 55149-1-AP
anti-PCNA	Santa Cruz Biotechnology	Cat# sc-56

Supplementary Table 4. Oligonucleotide sequences of qPCR primers used in this study

Gene	Primer sequence
MRE11	Forward: 5'-GGGGCAGATGCACTTTGTG-3'
	Reverse: 5'-GAAGCAAAACCGGACTAATGTCT-3'
RAD50	Forward: 5'-TAAGTGTGCAGAAATTGACCGAG-3'
	Reverse: 5'-GACGTACCTGCCGAAGTGTT-3'
RAD51	Forward: 5'-CGAGCGTTCAACACAGACCA-3'
	Reverse: 5'-GTGGCACTGTCTACAATAAGCA-3'
NBS1	Forward: 5'-CACTCACCTTGTCATGGTATCAG-3'
	Reverse: 5'-CTGCTTCTTGGACTCAACTGC-3'
BRCA1	Forward: 5'-ACCTTGGAACTGTGAGAACTCT-3'
	Reverse: 5'-TCTTGATCTCCCACACTGCAATA-3'
BRCA2	Forward: 5'-TGCCTGAAAACCAGATGACTATC-3'
	Reverse: 5'-AGGCCAGCAAACTTCCGTTTA-3'
OGG1	Forward: 5'-ATGGGGCATCGTACTCTAGC-3'
	Reverse: 5'-CTCCCTCCACCGGAAAGAT-3'
APE1	Forward: 5'-CAATACTGGTCAGCTCCTTCG-3'
	Reverse: 5'-TGCCGTAAGAAACTTTGAGTGG-3'
FEN1	Forward: 5'-CACCTGATGGGCATGTTCTAC-3'
	Reverse: 5'-CTCGCCTGACTTGAGCTGT-3'
XRCC1	Forward: 5'-TCAAGGCAGACACTTACCGAA-3'
	Reverse: 5'-TCCAACTGTAGGACCACAGAG-3'
POLβ	Forward: 5'-CCAGTGGTGACATGGATGTTC-3'
	Reverse: 5'-TGCTCCACAACCTGATGTAAC-3'
MPG	Forward: 5'-TAATGGCACAGAACTCCGAGG-3'
	Reverse: 5'-CATGCAGAAGTACATGCCGTA-3'
XPA	Forward: 5'-CCAGGACCTGTTATGGAATTTGA-3'
	Reverse: 5'-GCTTCTTGACTACCCCAAACTTC-3'
XPC	Forward: 5'-CTTCGGAGGGCGATGAAAC-3'
	Reverse: 5'-TTGAGAGGTAGTAGGTGTCCAC-3'
ERCC1	Forward: 5'-CTACGCCGAATATGCCATCTC-3'
	Reverse: 5'-GTACGGGATTGCCCCTCTG-3'
ERCC2	Forward: 5'-AGAAGGTGATTGAAGAGCTTCG-3'
	Reverse: 5'-ACCTCAGGGTGAATACACAAGT-3'
ERCC6	Forward: 5'-CGGTTCCTATACCCACAAAAAGG-3'
	Reverse: 5'-GCCAGTCATACCTGCTAATGTC-3'
PCNA	Forward: 5'-CCTGCTGGGATATTAGCTCCA-3'
	Reverse: 5'-CAGCGGTAGGTGTCGAAGC-3'
Ku70	Forward: 5'-TTGCTTCTGCCTAGCGATACC-3'
	Reverse: 5'-AAACCTGGATCATCAAACCGTT-3'
Ku80	Forward: 5'-GCACTGACAATCCCCTTTCTG-3'
	Reverse: 5'-TCAATGTCCTCCAGCAAATCAAA-3'
DNAPK	Forward: 5'-CTGTGCAACTTCACTAAGTCCA-3'
	Reverse: 5'-CAATCTGAGGACGAATTGCCT-3'

MGMT	Forward: 5'-TTTTCCAGCAAGAGTCGTTCAC-3'
	Reverse: 5'-GGGACAGGATTGCCTCTCAT-3'
MSH1	Forward: 5'-CAACAAGTCTGACCTCGTCTTC-3'
	Reverse: 5'-CCGGGAATCTGTACGAACCAT-3'
MSH2	Forward: 5'-CACTGTCTGCGGTAATCAAGT-3'
	Reverse: 5'-CTCTGACTGCTGCAATATCCAAT-3'
CHK1	Forward: 5'-ATATGAAGCGTGCCGTAGACT-3'
	Reverse: 5'-TGCCTATGTCTGGCTCTATTCTG-3'
ATM	Forward: 5'-TTGATCTTGTGCCTTGGCTAC-3'
	Reverse: 5'-TATGGTGTACGTTCCCCATGT-3'
ATR	Forward: 5'-TCCCTTGAATACAGTGGCCTA-3'
	Reverse: 5'-TCCTTGAAAGTACGGCAGTTC-3'
